# Evaluation of Ochratoxin Recognition by Peptides Using Explicit Solvent Molecular Dynamics

**DOI:** 10.3390/toxins9050164

**Published:** 2017-05-13

**Authors:** Aby A. Thyparambil, Ingrid Bazin, Anthony Guiseppi-Elie

**Affiliations:** 1 Center for Bioelectronics, Biosensors and Biochips (C3B), The College of Engineering, Texas A&M University, College Station, TX 77843, USA; athypar@tamu.edu; 2 Department of Biomedical Engineering, 5045 ETB, The Dwight Look College of Engineering, Texas A&M University, College Station, TX 77843, USA; 3 Ecole des mines d'Ales, institut Mines, Telecom, 6 avenue de Clavieres, Ales cedex30319, France; ingrid.bazin@mines-ales.fr; 4 ABTECH Scientific, Inc., Biotechnology Research Park, 800 East Leigh Street, Richmond, VA 23219, USA

**Keywords:** mycotoxin recognition, ochratoxins, peptide, molecular dynamics, NFO4, toxins, Markov state model, solvation penalty, binding free energy, biased exchange metadynamics

## Abstract

Biosensing platforms based on peptide recognition provide a cost-effective and stable alternative to antibody-based capture and discrimination of ochratoxin-A (OTA) vs. ochratoxin-B (OTB) in monitoring bioassays. Attempts to engineer peptides with improved recognition efficacy require thorough structural and thermodynamic characterization of the binding-competent conformations. Classical molecular dynamics (MD) approaches alone do not provide a thorough assessment of a peptide’s recognition efficacy. In this study, in-solution binding properties of four different peptides, a hexamer (SNLHPK), an octamer (CSIVEDGK), NFO4 (VYMNRKYYKCCK), and a 13-mer (GPAGIDGPAGIRC), which were previously generated for OTA-specific recognition, were evaluated using an advanced MD simulation approach involving accelerated configurational search and predictive modeling. Peptide configurations relevant to ochratoxin binding were initially generated using biased exchange metadynamics and the dynamic properties associated with the in-solution peptide–ochratoxin binding were derived from Markov State Models. Among the various peptides, NFO4 shows superior in-solution OTA sensing and also shows superior selectivity for OTA vs. OTB due to the lower penalty associated with solvating its bound complex. Advanced MD approaches provide structural and energetic insights critical to the hapten-specific recognition to aid the engineering of peptides with better sensing efficacies.

## 1. Introduction

**Figure 1:**
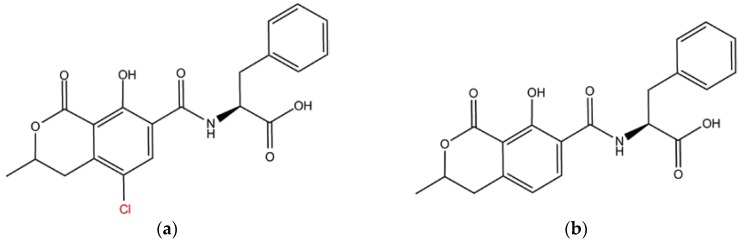
Chemical structure of (**a**) ochratoxin-A (OTA) and (**b**) ochratoxin-B (OTB). The structural analogues differ in the ‘Cl’ moiety, which is highlighted in red.

Ochratoxins are a group of chemically related mycotoxins produced by storage fungi like *Aspergillus ochraceus* and *Penicillium verrucosum* [1,2]. Structurally, these low-molecular-weight chemicals are polyketides with a dihydro-isocoumarin moiety linked to β-phenylalanine [1]. Though at least three different structural variants of ochratoxin are known to naturally occur, only ochratoxin-A (OTA) and its non-chlorinated analogue, ochratoxin B (OTB) are found prevalently in food, animal feed, and beverages (Figure 1) [1,3]. Of these two ochratoxins, OTA is more toxic [1]. OTA has been known to bind with high affinity to serum proteins and prolonged exposure has been linked with longer persistence in serum and tissues with implications for endocrine disruption, nephrotoxicity, genotoxicity, and increased cancer risks [1,2,3,4]. One approach to mitigate the health risks posed by OTA is to impose strict regulatory limits on the OTA levels in food matrices to control its bioaccumulation in the consumer. Currently, antibodies provide the specificity and sensitivity required for monitoring OTA levels (limit of detection <2.5 nM) in food, animal feed, and beverages [5,6,7]. Antibodies are widely used in immunoaffinity columns, enzyme-linked immunosorbent assays (ELISAs), test strips, and various biosensor formats [5,6,7]. However, the limited storage time, and sensitivity to operational processes (like buffers, salts, inhibitors, and others) severely shortens the service life and undermines the high production costs associated with the development of antibodies against haptens like OTA. Concerned producers and regulatory bodies such as the Regulatory Commission of the European Community [8] have been seeking new, faster, more convenient, and less expensive methods of real-time detection and/or monitoring of OTA [9].

Peptides and oligonucleotide-based aptamers are an attractive alternative to antibodies [6,10]. Peptides and aptamers are robust, more stable in a wide range of buffer solutions, and less prone to activity losses. More importantly, both peptides and aptamers are very cost-effective to produce as they can be rapidly generated to virtually any hapten target, including those that are toxic or of low immunogenicity. Because of these benefits, a variety of platforms based on immobilized peptides and aptamers have been developed against OTA during the last decade [6,10,11,12]. For example, Giraudi et al. were the first to report a hexapeptide receptor, raised using phage display libraries, for OTA targeting [13]. Heurich et al. later reported peptide receptors with improved affinity to OTA by identifying an octamer and a 13-mer using computational modeling approaches and validated the peptides using gravimetric techniques [14]. Recently, Bazin et al. designed and chemically synthesized a peptide, NFO4 (12 amino acids long), from a mimotope simulating oxidoreductase, and assessed the affinity of the peptides using ELISA [15]. However, none of the reported affinities and selectivity estimates for immobilized peptides to OTA have mimicked the sensing efficiencies of immobilized antibodies [7,16]. In contrast, immobilized systems based on guanine–cytosine-rich oligonucleotide aptamers have shown selectivity to OTA that is either comparable or better than some of the antibodies [6,10,11]. Some of these studies have also demonstrated that immobilization conditions such as pH, type of surface, recognition molecule density as well as other effects can promote, inhibit or have no effect on the in-solution affinities and selectivity of the biological recognition molecule [11,17]. In such circumstances, a cost-effective tool that aids the direct comparison of the in-solution binding capabilities of the recognition molecules is highly desired. Molecular Dynamics (MD) Simulation can potentially be that tool. 

Within a solvent, peptides are dynamic and exist in an ensemble of conformational states [18,19,20,21]. Of these conformation ensembles, haptens bind with high affinity to the most suitable peptide configuration or pose while weakly interacting with other peptide configurations. Consequently, peptide–hapten systems that bind with high affinity would shift the population distribution at equilibrium favoring the bound pose, while the low-affinity peptide–hapten poses would skew the equilibrium towards an unbound pose [20,22]. In essence, a peptide’s hapten binding properties, including its affinity and specificity, can be thoroughly assessed or even engineered by considering the population distribution and redistribution of a peptide’s conformational states, in tandem with the structural–energetic characteristics of the bound poses. Unfortunately, a detailed characterization of a peptide’s diverse conformations or its dynamic properties is often beyond the capabilities of routine biophysical techniques like equilibration dialysis or surface plasmon resonance. Even with the advances in computer architecture, regular MD approaches alone are inept at generating thermodynamically favored conformations of peptide–hapten complexes or replicating the kinetic process involved in peptide–hapten binding [19,22]. For example, regular MD simulations typically capture biophysical phenomenon occurring on the order of hundreds of nanoseconds, while the unbinding kinetics of a peptide–hapten system with high affinity would involve time scales often exceeding several hours. To overcome these limitations, the authors propose the use of an MD approach that involves the tandem application of an accelerated conformational sampling and predictive modeling approach. The accelerated conformational sampling would generate a series of kinetically relevant conformational states while predictive modeling will be used to estimate the transition kinetic rates involved in these conformation ensembles.

Accelerated sampling approaches are a special kind of MD simulations that unlike regular MD approaches skew the energy barrier separating the lower free energy conformational states by either introducing thermal energy (like replica exchange MD (REMD)) or introducing time dependent bias-potential (biased exchange metadynamics (BEMD)) [23,24,25]. Such sampling approaches have previously shown great promise in generating experimentally relevant biomolecular conformations [25]. REMD uses elevated temperatures to provide the thermal energy necessary to rapidly cross the energy barriers and samples the relevant peptide configurations. In contrast, BEMD involves sampling at the same temperature but explores the conformational landscapes of the biomolecule by introducing a time-dependent bias potential as a function of the chosen collective variables (CV) [26]. The CVs are explicit functions that control the atomic coordinates of the biomolecule and describe the slow-order transition from its initial conformation to metastable conformation. Each replica in a BEMD simulation is biased with a time-dependent bias-potential acting on one or two different CVs. After a certain time, exchanges between different pairs of replicas are attempted using the Metropolis scheme. If the exchange is accepted, the CV of replicas involved will be also exchanged. Thus, the exploration of the conformational landscape that was initially biased in the direction of the first CV continues its exploration biased by the second CV (and vice versa) [25,27]. In this manner, many different conformations can be simultaneously explored by BEMD simulation as opposed to a regular MD simulation. Recent studies have further shown that with optimal CV definitions, BEMD approaches are more efficient than REMD for conformational sampling [25,28].

**Figure 2:**
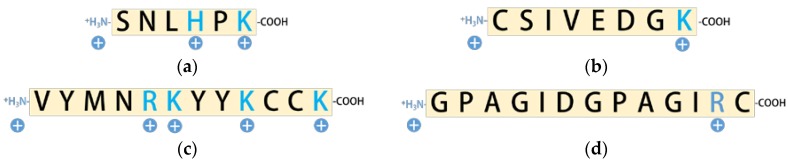
One-letter amino acid sequences of peptides: (**a**) hexamer (SNLHPK); (**b**) octamer (CSIVEDGK); (**c**) NFO4 (VYMNRKYYKCCK); and (**d**) 13-mer (GPAGIDGPAGIRC), currently developed for OTA-specific capture and recognition. The N-termini of the peptides were represented as –NH_3_^+^ and C-termini of the peptide were represented as –COOH. The likely protonation states of the peptides in a wine-like solution condition were shown with the positively charged amino acids depicted in blue and uncharged amino acids colored in black.

Nevertheless, the time period in any accelerated sampling simulation do not represent the actual duration of continuous dynamics but rather the amount of sampling that is conducted. In other words, while the sampling approaches determine the ensemble average of states, the trajectories do not necessarily represent the actual dynamic time sequence of events and therefore limits any direct kinetic estimates of the biomolecular events from such simulations. In such a scenario, predictive modeling approaches, such as Markov State Model (MSM) analysis, can greatly aid the prediction of dynamic properties involved in peptide–hapten binding, e.g., binding free energy, dissociation constant, and ‘*on*’ and ‘*off*’ rates [22,29,30,31]. MSM approaches involve the linear transformation of the configuration space into collective coordinates that are then sorted by ‘*slowness*’ into discrete states, such that the transitions within each discrete state are fast but the transitions across different states are slow. The transition probabilities between each of the states would then provide a kinetic estimate of these transitions and provide insights into the affinity and selectivity of a peptide. Therefore, the objective of the current study was to assess the sensing efficacies, specifically the affinity and selectivity of four different peptides known for their molecular recognition of OTA (Figure 2) using BEMD and MSM approaches. Control studies were also carried out with OTB, to better understand the fundamental interactions critical to improving the affinity and selectivity of peptides to OTA. All binding studies were carried out in an explicit solvent modeled environment that mimics the solution pH (3–4) of wine. The outlined methods could aid the screening and eventually the engineering of peptide systems with desired ochratoxin recognition efficiency. Lastly, the applicability of the outlined methods will not be limited to the current study alone but could be extended to virtually any peptide–hapten systems.

## 2. Results and Discussion

### 2.1. Conformational Preferences of Peptide in Solution

**Figure 3:**
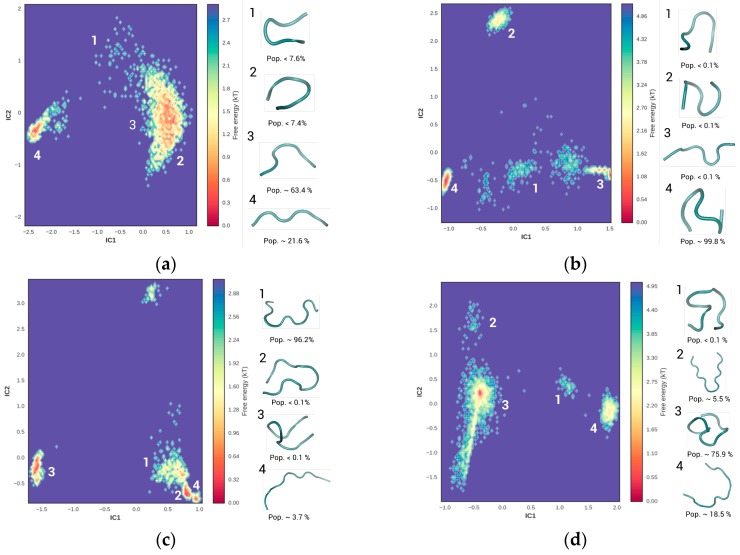
The free energy landscape and the four lowest energy configurations of the four peptides: (**a**) hexamer (SNLHPK); (**b**) octamer (CSIVEDGK); (**c**) NFO4 (VYMNRKYYKCCK); and (**d**) 13-mer (GPAGIDGPAGIRC) are illustrated. The equilibrium distribution of the folded peptide poses was provided as “Pop.” The scale bar indicates the basin depth of each contour feature, with bars towards the blue regime indicating the least favorable peptide configuration while the red regime indicates the most favorable peptide configuration. IC1 (*x*-axis) and IC2 (*y*-axis) represents the dimensional projection along the top two components of the IC analysis.

Multiple conformations of the unbound peptide were explored using BEMD simulations and its population distributions at equilibrium were identified via MSM analysis [32,33]. All simulations were initiated from a fully extended peptide conformation and were carried out until the system equilibrated (Supplementary Figure S1). Peptide conformations were then sorted using the contact distance between a pair of heavy atoms as the metric. Independent component (IC) analysis on the feature space identified five slowest dimensions that contributed to over 95% of the kinetic variance in a peptide’s configurational space. The configurational space was subsequently discretized into four macrostates based on the frequency of backbone contact. Figure 3 represents the free energy surface (FES) along the projection of the two major IC components (represented by IC1 and IC2). Table 1 summarizes the structural and thermodynamic preferences of each of the four systems. Based on peptide backbone contacts, the top four macrostates represented as ‘1’, ‘2’, ‘3’, and ‘4’ were identified. For each discrete macrostate, the water density surrounding the peptide (Supplementary Figure S2), the internal contact map (Supplementary Figure S3), the H-bonding profile (Supplementary Figure S4), and the 2° structure content (Supplementary Figure S5) were characterized. 

#### 2.1.1. Hexamer (SNLHPK)

Figure 3a represents the FES of hexamer folding, when the configurational space of the peptide resolved by IC1 and IC2 components were projected onto a 2D space. Two distinct basins along with several diffused islets were observed. Representative peptide structures from each basin are shown in the adjacent panel. For example, Macrostate 1 was representative of the diffused basin in the top portion of the graph (Basin 1). Similarly, Macrostates 2 and 3 were sampled from the larger basin (Basin l) on the right edge of the graphs. Macrostate 4 was sampled from the basin on the middle-right region of the graph (Basin 4). While Basin l accounts for over 70% of the peptide configurations, 22% of the sampled configurations were accounted for by Basin 4. The diffused islets that were distributed in between these two major basins (Macrostate 1) account for the remaining 8% of the peptide configurations. Each of the basins had a distinct contour feature. While the contours of Basin l were larger and wider than the other basins, it was roughly the same depth as Basin 4 (~2.7 kT). The energy barrier separating the conformations sampled from Basin 1 were relatively small (<1.0 kT), and is likely a conformation intermediate of the macrostates represented in Basin l and Basin 4. Structural analysis of the sampled peptide conformations indicated a general preference towards a random coil conformation (see Supplementary Figure S5a) with at least two inter-peptide H-bonds between the residues Ser-1, Asn 2, His 4 and Lys-6 (see Supplementary Figure S3a) due to its close proximity. Supplementary Figure S4a provides the average H-bonds profile within each discrete macrostate. Interestingly, an inverse correlation was observed in the frequencies of intra-peptide H-bonds and peptide–solvent H-bonds. The trend suggests that the disruption in the peptide–solvent might be compensated by peptide–peptide H-bonds (see Supplementary Figure S4a).

#### 2.1.2. Octamer (CSIVEDGK)

Figure 3b shows at least six distinct basins within the FES representing the octamer folding. Each of the basins was of relatively equal size, distinct, and separate from each other. Macrostate 1 was sampled from the diffused basin on the lower middle region of the graph (Basin 1). Similarly, Macrostates 2 and 3 were representative of the basins on the top (Basin 2) and lower right (Basin 3) regions in the graph. Macrostate 4, which that accounts for >99% of the sampled peptide conformations, was sampled from the basins on the lower left edge of the graph (Basin 4). The narrow conformational distributions also indicated a conformational bias in octamer folding. Energetically, the basins with the most favorable conformations were ranked as: Basin 4 (most favorable, >4.9 kT) >Basin 3 > Basin 2 > Basin 1 (least favorable, <1 kT). Structural differences were also observed among the peptides sampled from different basins. While all of the peptides generally preferred a random coil (see Supplementary Figure S5b), its packing was different in each macrostate (see Supplementary Figure S2b). In a majority of the sampled peptides (i.e., Macrostate 4), water density surrounding the peptide was relatively low, as evidenced from the lower radial distribution function (RDF) values at distances <0.5 nm (see Supplementary Figure S2b). Similarly, these peptides also showed a higher tendency to reduce peptide–solvent H-bonding while maximizing the number of inter-peptide H-bonds (see Supplementary Figure S4b). Both of the above results together suggest that the octamers generally prefer to maximize H-bonding within the peptide segments (between Ser2, Ile3, Glu5, Asp6, Gly7, and Lys8) while minimizing H-bonding with the surrounding solvent (see Supplementary Figures S3b and S4b).

#### 2.1.3. NFO4 (VYMNRKYYKCCK)

Figure 3c shows at least three distinct energy basins within the FES representing the folded conformations of NFO4. Macrostates 1, 2, and 4 were sampled from a single basin on the lower right corner of the graph that accounted for >99% of the sampled peptide conformations (Basin l). In contrast, Macrostate 3 represented the peptide conformations that were sampled from the basin in the lower left corner of the graph (Basin 3). Basin 3 was isolated and barely accounted for 0.01% of the sampled peptide conformations. Nevertheless, the energy wells of Basin l and Basin 3 were rugged with the presence of multiple local minima, with the energy barrier between these minima often being less than 0.1 kT. The low energy barriers between each well may facilitate the quick conformational transitions from one macrostate to another, enabling frequent conformational switching. Consequently, at equilibrium, most of the peptide configurations preferred a more open/extended conformation that maximized H-bonding with the solvent while minimizing the intra-peptide H-bonding (see Supplementary Figure S4c).

#### 2.1.4. 13-mer (GPAGIDGPAGIRC)

Figure 3d shows four distinct energy basins within the FES representing the folded conformations of 13-mer. Macrostates 1 and 2 represent the diffused basins in the middle (Basin 1) and top left region (Basin 2) of the graph. Similarly, Macrostates 3 and 4 were representative of the basins on the left (Basin 3) and right regions (Basin 4) of the graph. Basins 3 and 4 together account for >90% of the sampled peptide configurations. However, unlike the contour feature of Basin 4, the contour feature of Basin 3 was narrower and deeper (energy difference >5 kT) than any other basin. While all the peptides sampled were generally unstructured (see Supplementary Figure S5d), the configurations sampled from Basin 3 were the most compact (evident from R_g_ values, data not shown), less solvated (see Supplementary Figure S2d), and generally stabilized by an average of six intra-molecular H-bonds between the residues Ala3, Gly4, Ile5, Asp6, Gly7, Arg12, and Cys13 (see Supplementary Figure S4d) due to its closer proximity (<0.5 nm).

In short, the combined assessments of the above results suggest that the projection of IC components on a 2D graph is an effective approach to identify the most populated/favored peptide configurations. The peptides in the current study showed a general preference for a random/coil like structure but there were significant differences in the packing preference of each of these peptides. Both the octamer and 13-mer preferred configurations tend to maximize internal H-bonding while minimizing the H-bonding with the solvent. In contrast, the hexamer and NFO4 configurations favored H-bonding with the solvent while minimizing the internal H-bonds. This behavior was not unexpected, as among all the peptides octamer and 13-mer were relatively more hydrophobic than NFO4 and hexamer. For the binding simulation, irrespective of the peptide’s conformational distribution, binding simulation was initialized from all four conformations that were identified in Figure 3 to avoid any conformation bias in the binding simulation runs.

### 2.2. Structural and Energetic Characteristics Influencing the Peptide Affinity and Selectivity to Ochratoxins 

**Figure 4:**
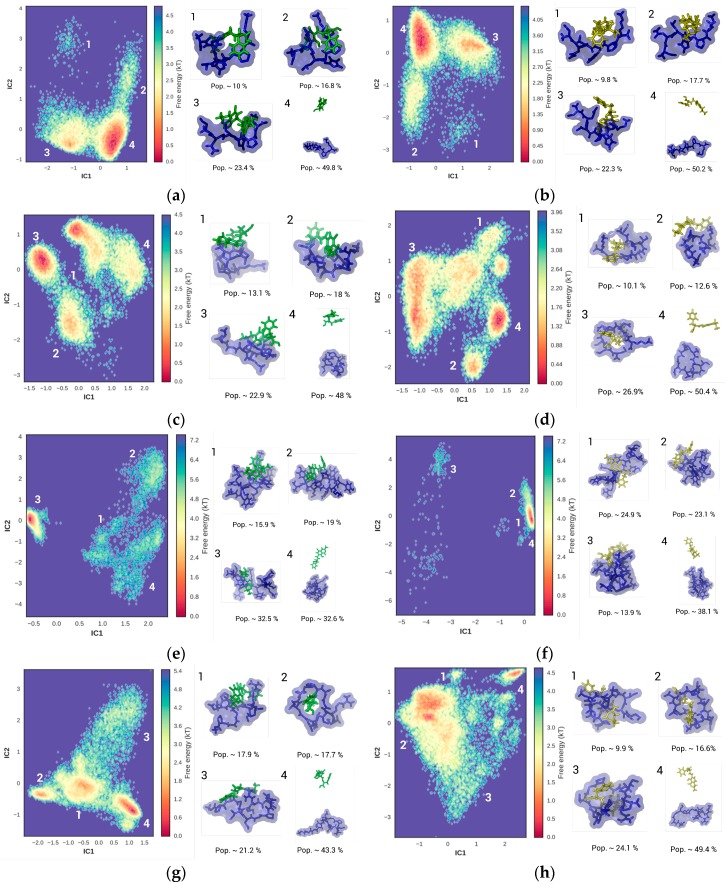
The free energy landscape, peptide–hapten configuration, and equilibrium distribution associated with (**a**,**b**) hexamer; (**c**,**d**) octamer; (**e**,**f**) NFO4 and (**g**,**h**) 13-mer when bound to (**a**,**c**,**e**,**g**) OTA (represented in green) and (**b**,**d**,**f**,**h**) OTB (represented in yellow) are shown. Peptides are depicted with the molecular surface and side-chains of the constituent amino acids in the peptide are colored in blue. The equilibrium distributions of each bound pose were provided as Pop. The scale bars indicate the FES estimates based on the frequency of contacts within a contact distance of 0.5 nm. The bluer regions within the FES map were associated with reduced contact frequency and redder region were representative of higher contact frequency. Macrostates 1, 2, and 3 represent the bound state of the peptide–hapten complex while Macrostate 4 represents the unbound state of the peptide. IC1 (*x*-axis) and IC2 (*y*-axis) represent the dimensional projection along the top two components of the IC analysis.

Binding affinities and equilibrium population distributions of peptide when bound to OTA and OTB were predicted using BEMD and MSM analysis. Simulations were initialized with the peptides and ochratoxins in dissociated states (i.e., distance >2.5 nm), and were carried out until system equilibration. Each of the four systems showed an average exchange rate of ~0.26. Using the nearest neighbor heavy-atom contacts within the peptide and ochratoxins as input features, the trajectories were projected onto the three slowest coordinates using IC analysis. Figure 4 shows the FES associated with peptide (hexamer, octamer, NFO4, and 13-mer) binding to OTA and OTB, and were obtained by the projection of top two IC components. Table 2 summarizes the structural and thermodynamic preferences of each of the four systems. The average binding affinities and the statistical error associated with these binding estimates were obtained by block averaging the data over 20 ns. In this study, peptides were considered bound when the haptens were within 0.5 nm of the peptide. Based on the peptide–hapten contacts, the top four macrostates, represented as ‘1’, ‘2’, ‘3’, and ‘4’ were identified. Macrostates 1, 2, and 3 represent the bound state of the peptide–hapten complex and Macrostate 4 represents the unbound state of the peptide. For each of the discrete macrostates, the water density surrounding the complex (Supplementary Figure S6), residue flexibility (Supplementary Figure S7), 2° structure (Supplementary Figures S8 and S9), contact map (Supplementary Figures S10 and S11), H-bonding profile (Supplementary Figures S12 and S13), the energetic components (Supplementary Tables S1 and S2), and residue-wise energetic contribution (Supplementary Tables S3–S6) were assessed to thoroughly characterize the process of peptide binding to ochratoxins. As the binding energy estimates determined in this study were based on the definitions provided in Equation (1), the bulk of the discussion focuses on the structural and energetic characteristics of the most dominant population.

#### 2.2.1. Hexamer–OTA/OTB

Figure 4a,b shows the FES that corresponds to hexamer binding to OTA and OTB, respectively. Both hexamer–OTA and hexamer–OTB systems generated a FES with two major basins —a super basin that was sampled by three distinct macrostates and a diffused basin that was sampled by a single macrostate. The super basins account for >80% of the sampled configurations. The contours of both super basins featured a broad and deep well (>4 kT) that were flanked by two shallower wells on either side. In both systems, Macrostate 1 sampled the diffused basin and Macrostates 2, 3, and 4 sampled the super-basin. Roughly 50% of the peptide configurations were categorized as the bound state (Macrostates 1, 2, and 3), while the remaining 50% of the peptide configurations were categorized as the unbound state (represented by Macrostate 4). Within the bound complex of both the systems, the most populated and the most favored pose was represented by the meta-stable state 3 (well depth >3 kT). Closer examination of the structural and interaction network of the hexamer–hapten configurations provided a more detailed insight into the underlying interactions. The unbound hexamer was generally unordered (100% random coil like structure) with a dense water layer surrounding the peptide (Supplementary Figures S6a,b, S8a and S9a). The unbound peptides favored H-bonding with the surrounding solvent and minimized internal H-bonding or contact while being generally less flexible (Supplementary Figures S6a,b, S12a, and S13a). Similarly, in the unbound state, haptens were hydrated and favored H-bonding with the solvent while minimizing hapten–hapten H-bonding. Post-binding, the solvent density surrounding the peptide (Supplementary Figure S6a,b) and haptens were reduced and was accompanied by reduced peptide–solvent H-bonds, reduced hapten–solvent H-bonds and increased frequency of peptide–hapten H-bonding (Supplementary Figures S12a and S13a). The binding process however, did not induce 2° structural shifts (Supplementary Figures S8a and S9a) or alter the internal H-bonds or contact network of the hexamer. In the bound state, OTA was generally in contact (<0.35 nm) with His-4 and Lys-6, while OTB was in contact with the entire set of residues barring Ser-1 (Supplementary Figures S10a and S11a). Using the discrete macrostates identified via MSM as trajectories, the energetic components involved in the ochratoxin binding process (Supplementary Tables S1 and S2) were identified. It is important to note that the binding energetic components were not explicitly treated as enthalpic or entropic components, but rather a cumulative function of the non-bonded interactions (like electrostatic and van der Waals interactions), solvation penalty (associated with solvating the polar and non-polar groups within the bound complex) and conformational entropy. While the conformational entropy in the system was estimated using quasi-harmonic analysis approach, the non-bonded interactions and solvation penalties were estimated using the MM-PBSA method, as outlined in Section 2.2.4. Supplementary Tables S1 and S2 provide the breakdown on the energetic components involved in the hexamer–hapten interactions. The non-bonded interactions were evidently stronger in the hexamer-OTB system than in hexamer-OTA system. However, the penalties associated with the solvation and configurational entropies significantly undermined the enthalpic benefit of the non-bonded interactions of hapten with multiple residues. Consequently, the in-solution affinities of the hexamer to both the ochratoxins were relatively similar.

#### 2.2.2. Octamer–OTA/OTB

Figure 4c,d shows the FES that correspond to octamer binding to OTA and OTB, respectively. The FES for octamer binding with OTA indicates at least two major basins—a super-basin encompassing Macrostates 2 and 3 on the left side of the graph, and a basin representing Macrostate 4 in the top right of the graph. Macrostate 1 sampled the diffused region bridging the two major basins. The bound poses of the octamer–OTA complex account for 52% of the sampled peptide configurations and were majorly sampled from the super-basin in Figure 4c. The remaining 48% of the octamer configurations represent the unbound peptide configuration and were sampled from Basin 4. The contours of the basins indicated several energy wells of varying depths with the deepest well located in Basin 3 (>4 kT), followed by a shallower well in Basin 2 and Basin 4. Similarly, at least three basins were observed in the FES of octamer–OTB complex (Figure 4d)—a super-basin (encompassing Macrostates 1 and 3) that stretched from the left to the top right of the graph, a basin (encompassing Macrostate 4) with a deep energy well (Basin 4) in the bottom right corner of the graph, and a shallow basin (encompassing Macrostate 2) in the lower middle (Basin 2) region of the graph. Overall, Macrostates 1, 2, and 3, which represent the bound octamer–OTB complex, account for <50% of the sampled peptide configurations. The remaining 50% were represented by the unbound Macrostate 4. Among the bound complexes, meta-stable state 3 (~4.5 kT for OTA, and ~3 kT for OTB) was more populated (octamer-OTA—23%; octamer-OTB—27%), and more energetically favored. With regard to the structural characteristics, the unbound octamers were generally unordered (100% random coil) with a dense water layer surrounding the peptide (Supplementary Figure S6c,d). Unbound haptens also favored H-bonding with the solvent over forming internal H-bonds (Supplementary Figure S12b and Figure S13b). However, unlike in the hexamer system, the unbound octamer was stabilized by both an internal H-bonding network and solvent-mediated H-bonds (Supplementary Figures S10b, Figure S11b, Figures S12b, and S13b). Subsequent to binding, all macrostates showed a reduced solvent density surrounding the peptide (Supplementary Figure S6c,d). The desolvation process leads to a reduced number of peptide–solvent H-bonds, a reduced number of hapten-solvent H-bonds, and an increased number of peptide–hapten H-bonds (Supplementary Figures S12b and S13b). However, hapten binding to the octamer did not induce 2° structural shifts, alter the internal contacts, or alter the peptide’s flexibility (Supplementary Figures S7c,d, S8b, S9b, S10b, and S11b). In some of the bound complexes of octamer with OTA (specifically in Macrostates 1 and 3 of Supplementary Figure S12b), peptide–hapten H-bonding were accompanied by a disruption in the intra-peptide H-bond network following the desolvation of peptide and haptens. In Macrostate 3, OTA was frequently in contact with three of the eight residues (Asp-6, Gly-7, and Lys-8), while OTB was in contact with all the residues (Supplementary Figures S10b and S11b). This observation also complemented the high energetic contribution by the non-bonded interactions that were observed in Supplementary Tables S1 and S2. However, the relatively high penalties involved in solvation significantly undermined the affinity of these peptide systems to ochratoxins.

#### 2.2.3. NFO4–OTA/OTB

Figure 4e,f shows the FES corresponding to NFO4 binding to OTA and OTB, respectively. Figure 4e shows two major basins—a diffuse super-basin that spans the top right to lower right edges of the graph, and a narrow basin in the middle left region in the graph. Macrostates 1, 2, and 4 were sampled from the central, top right, and lower right regions of the super-basin, while Macrostate 3 was sampled from the basin in the middle left region of the graph. Similarly, NFO4 binding to OTB also showed two major basins (Figure 4f)—a diffuse basin that spans the top left to lower left region of the graph, and a narrow super basin in the middle right region of the graph. The narrow super-basin encompassed three macrostates, with Macrostate 1 sampling the deep well in the super-basin, Macrostate 2 sampling the shallower region in the super-basin, and Macrostate 4 sampling the diffuse region extending out of the narrow basin. In both systems, the bound poses account for >60% of the sampled configurations. Structural and energetic characterization of the macrostates provided additional insights into the mechanistic differences involved in NFO4 binding to each of the ochratoxins. The structure of unbound NFO4 was generally ordered with a prominent 2° structural features (50% sheets and 50% random coil like structure) and was stabilized by numerous internal contacts including peptide–peptide H-bonding (Supplementary Figures S8c, S9c, S10c, S11c, S12c, and S13c) and peptide–solvent contacts with the dense water layer surrounding the peptide (Supplementary Figure S6e,f). It was additionally observed that the terminal ends of the unbound peptide were relatively flexible when compared to the interior segments of the peptide (Supplementary Figure S7e,f). Post-binding, configurations sampled by Macrostate 3 of the NFO4–OTA complex and Macrostate 1 of the NFO4–OTB complex were the most populated states and more energetically favored. Within these macrostates, peptides desolvated and switched from an ordered structure to a predominantly random/coil like structure (Supplementary Figures S8c and S9c) with a significant drop in internal contacts. The desolvation of peptide and hapten promoted H-bonding and other external contact with the ochratoxins (Supplementary Figures S12c and S13c). Interestingly, NFO4 binding to ochratoxins increased the flexibility of the peptide, with the terminal ends of the peptide being more flexible than the interior segments (Supplementary Figure S7e,f). Considering that both peptide systems underwent a similar extent of disruption in their internal networks and formed a similar extent of peptide–ochratoxin contacts, a major difference was observed in the water density surrounding the peptides post-binding to the ochratoxins. Initially, the water densities surrounding the unbound peptides were generally high. However, post-binding, the NFO4–OTA system showed reduced solvent density, while NFO4–OTB systems showed higher solvent density (Supplementary Figure S6e,f). This difference was further reflected in the peptide–solvent’s H-bonding profile (Supplementary Figures S12c and S13c). Another critical difference was observed in the site of ochratoxin interaction. Within the most favorable pose of NFO4–ochratoxin complexes, OTA generally preferred interaction with residues nearer to the N-terminus of NFO4 (Tyr-2 and Asn-4), while OTB preferred to interact with the residues (Lys-12) nearer to the C-terminus of the peptide (Supplementary Figures S10c and S11c). Evidently, although the van der Waals energetic component dominated the OTA system and the electrostatic component dominated the OTB system, the non-bonded energetic components in the NFO4–OTA and NFO4–OTB complexes were quite similar (Supplementary Tables S1 and S2). The conformational transition and the increased flexibility of the bound complexes also increased the conformational entropic penalties and reduced NFO4’s overall binding affinity to ochratoxins. However, the solvation penalty was a more critical element influencing the NFO4 binding capabilities. Unlike other systems, the solvation penalties for both NFO4 complexes were significantly different. In fact, lower solvation penalties of NFO4–OTA complex improved NFO4’s overall affinity to OTA and, thereby, its selectivity to OTA.

#### 2.2.4. 13-mer–OTA/OTB

The unbound 13-mer was unordered with a prominent random coil like structure (100%) and its internal structure was stabilized by peptide–peptide H-bonding and H-bonding with the dense water layer surrounding the peptide (Supplementary Figures S6g,h, S8d and S9d). Additionally, it was observed that the terminal ends and middle segments of the peptide were relatively flexible compared to other interior segments (Supplementary Figure S7g,h). The 13-mer binding to OTA (Figure 4g) showed a single large super-basin encompassing all macrostates labeled 1 to 4. Among these macrostates, Macrostate 2 was sampled from the lower left region of the basin, while Macrostate 4 was sampled from the lower right region of the graph that includes a deep well region. Macrostates 1 and 3 were sampled from the diffuse region of the FES, with Macrostate 1 being sampled from the lower right region surrounding Basin 4 and Macrostate 3 was sampled from the region spanning the top right to the shallow wells in the lower middle region of the graph. Similarly, the FES of 13-mer bound to OTB also showed (Figure 4h) a super-basin and an isolated basin in the top right corner of the graph. Macrostates 1, 2, and 3 were sampled from the wells in the top left, shallower regions in the top middle, and the diffused regions of the super-basin, respectively. The isolated basin was sampled by Macrostate 4. In general, the bound complexes in both the systems account for >50% of the sampled configurations, among which Macrostate 3 was the most populated but not necessarily the most energetically preferred bound state. Post-binding, peptides did not undergo any major conformational shifts but some of the peptide’s internal contacts (not necessarily the H-bonds) were disrupted (Supplementary Figures S8d, S9d, S10d, and S11d). The RDF profile (Supplementary Figure S6g,h), along with H-bonding profile (Supplementary Figures S12d and S13d) suggests that OTA binding to 13-mer was driven by desolvation of both the peptide and OTA. The data also suggest that OTB binding to NFO4 was driven solely by the desolvation of OTB. Supplementary Tables S1 and S2 indicate that while non-bonded energetic components of the 13-mer–OTA and 13-mer–OTB complexes were relatively similar, the lower solvation penalty for the 13-mer–OTB complex and the higher conformational entropy for the 13-mer–OTA system together compromised the overall affinity and selectivity of the 13-mer systems to ochratoxins.

### 2.3. Validation of Results and Implications for Computer-Aided Design of Biosensing Platforms

Table 3 summarizes the predicted standard state binding free energies (ΔG°) and dissociation binding constants (K_D_) for different peptide–hapten systems in a wine-like solution environment. Experimentally derived estimates for each of the peptide systems tested in the current study were also provided to assess the validity of the *in silico* predictions. Lastly, the binding constants and selectivity of the immobilized antibodies and DNA-based aptamers in Table 3 provide a benchmark to compare the binding capabilities of different biological recognition elements to OTA. 

Table 3 shows that, among all the peptides studied, the predicted in-solution affinities of NFO4 for both OTA and OTB were significantly better (based on ΔG°_OTB-PRE_, ΔG°_OTB-PRE_, K_D-OTA-Pred_ and Selectivity-Pred. estimates) than the other peptides. The predicted in-solution selectivity of NFO4 (~13) to OTA vs. OTB was also better than any other peptide. Lastly, the predicted affinities of other peptide systems (i.e., hexamer, octamer, and 13-mer) were statistically similar, with no visible selectivity to the ochratoxins. To validate the accuracy of these predictions, the K_D_-_OTA_-_Pred_ of the peptides were compared with the K_D_-_OTA_-_Expt_. In line with the MD predictions, experimental data also indicated superior in-solution NFO4 properties. However, the magnitudes of K_D-OTA-Pred_ were ~2 orders of magnitude higher than the K_D-OTA-Expt_ estimates, and could be attributed to variations in experimental settings or the force field limitations. Experimentally derived binding data were obtained using routine biophysical techniques (like SPR, HPLC) under environmental conditions that were unique to each study design [13,14,15]. In such studies, the in-solution dynamics may have been compromised by the routine experimental practices like peptide conjugation, bio-immobilization, or ochratoxin tagging, such as by conjugation with a larger enzyme, that are essential for the detection and monitoring of the analytes [6,11]. MD simulations are without these physicochemical constraints. A second major reason for the wide variation in the K_D_ estimate could be due to the limitations in the force field used in the current study. While the force field is intended to describe the interactions between each of the atomic/molecular components in the system, topology parameters describe the physicochemical and bond information of the atom/molecule. Although those force fields that capture the finer details in atomic/molecular interactions are more accurate and improve the overall quality of the prediction, the computational costs associated with such force-fields are prohibitively high. Also, the molecular topology of the ochratoxins was not readily available and was generated using an automated server, which may need further optimization to improve its accuracy. To balance the predictive accuracy and computational cost, semi-empirical force fields like GROMOS are often reliably applied in protein–ligand binding applications. Accordingly, MD simulations capture the general trend in molecular interactions but not necessarily the exact magnitude of these interactions [19]. Nevertheless, the overall data trends suggest a general qualitative agreement between the prediction and experimental results, based upon which the molecular processes influencing the peptide–hapten binding were considered accurate.

Binding energy was decomposed as a cumulative sum of three main energetic components—non-bonded energetic contributions, solvation penalty, and conformational entropy. Supplementary Tables S1 and S2 show that the contributions by non-bonded interactions were strong and similar in NFO4, octamer and 13-mer peptide systems, even though the relative contribution of electrostatic and van der Waals components towards the overall non-bonded interactions varied from system to system. In fact, the strong contributions by non-bonded interactions were corroborated by the *in silico* binding free energy estimates for octamer and 13-mer system in an implicitly solvent modeled environment [14]. In the case of the hexamer system, the non-bonded interactions were the weakest among all peptide systems and no significant differences were observed in its interaction with OTA and OTB. However, the system-to-system variations in the solvation penalty (specifically of the polar group) and conformational entropies critically influenced the overall hapten binding properties of the peptide. Desolvation of the peptides and ochratoxins is important to peptide–ochratoxin binding. Generally, the desolvation processes involves reorganization of solvent–solvent and solvent–solute interactions, and is accompanied by an increase in peptide and ochratoxin contacts, including an increase in the number of new peptide–ochratoxin H-bonds (Supplementary Figures S12 and S13). This behavior is expected as H-bonds require the desolvation of both donor and acceptor groups [37]. While the solvation penalty is expected to be minimal for the non-polar groups, these penalties were expected to be higher for the polar groups, especially for those amino acid groups that were involved in charge reinforced H-bond interactions with the haptens. Furthermore, among the two ochratoxins, the solvation penalty for OTA was expected to be lower than OTB, as chlorinated analogues are generally more hydrophobic than their non-chlorinated counterpart. Supplementary Tables S4–S8 show the residue level break-up of the interaction in each molecular system. Except for the NFO4–OTA system, the ochratoxins were more frequently in contact with the more polar residues in all systems. However, the OTA interactions with NFO4 were more localized to the N-terminus of NFO4, which was more hydrophobic than the other segments. Consequently, the overall solvation penalty was significantly lowered, thereby improving the NFO4’s affinity and selectivity to OTA. A final factor influencing the affinity and selectivity of the peptides was the conformational entropy. As the conformational shifts accompanied NFO4 binding to OTA and OTB, the entropic penalties involved significantly reduced the overall affinity without compromising the selectivity of the NFO4. However, in the case of the 13-mer, its affinity to OTA was lowered by the entropic penalty involved, thereby compromising the peptide’s overall selectivity to OTA. Thus, the current study demonstrates that in addition to non-binding interactions, other energetic components such as the solvation penalties and conformational entropies also contribute to the overall binding energy estimate and may be more significant. This inference is particularly important for high throughput screening of novel peptide receptors that utilize implicit solvent based computational platforms but do not account for solvent dynamics to improve the affinity and selectivity of the peptides. The current study clearly illustrates the limitations of such an approach.

Although explicit solvent-modeled MD simulations are computationally more expensive than implicit solvent-modeled systems, the former approach does provide a more accurate mechanistic insight into the hapten binding process and offers clues to engineer better hapten-sensing platforms that could match the affinities of antibodies while retaining the selectivity of the aptamer (Table 3). One approach to designing better sensing platforms is via the modulation of the overall energetic component of the system. For example, experimental studies on the immobilized hexamer on a wide range of substrates indicate that the peptide’s affinity to OTA on solid phase (K_D_ ~ 0.01 μM–1.00 μM) were drastically different from those in-solution (K_D_ ~ 29 μM) [17]. Such a shift may be due to the altered solvation penalty, as high solvation penalty was responsible for the lowered in-solution affinity of the hexamer to ochratoxins (see Supplementary Tables S1 and S2). That is, these observed differences may not be due entirely to steric constraints imposed by immobilization but rather the surface may have an active role in recognition. Another avenue for a rationally guided approach to engineer peptides with improved ochratoxin sensing could be to identify specific sites within the peptide for point mutation or controlled modification to control the ochratoxin binding properties. For example, a key residue responsible for the high polar solvation penalty in NFO4–OTB complex was Lys-12 (Supplementary Table S5b). However, in the case of the NFO4–OTA complex, the solvation penalty by Lys-12 was relatively low, and contributed minimally to the overall solvation penalty (Supplementary Table S5a). A point mutation or the elimination of charge by some modification of Lys-12 is likely to reduce or eliminate the OTA selectivity of NFO4.

## 3. Conclusions 

Advanced sampling and predictive modeling were used for the in-solution characterization of the affinity and selectivity of four unique peptides (a) hexamer (SNLHPK); (b) octamer (CSIVEDGK); (c) NFO4 (VYMNRKYYKCCK); and (d) 13-mer (GPAGIDGPAGIRC) known for their molecular recognition of ochratoxins. *In silico* simulations were done in a wine-like solution environment because of the commercial and health relevance of ochratoxin contamination of wine. The *in silico* framework outlined in the current study facilitated structural and thermodynamic characterization of the systems in great detail that are otherwise not obtainable using routine biophysical techniques or regular MD approaches. Results show that while non-bonded interactions of octamer, NFO4, and 13-mer with the ochratoxins contributed significantly to the overall binding energetics, the lower solvation penalty associated with the NFO4–OTA complex was the most critical consideration responsible for the superior in-solution properties of NFO4. Results also indicate that modeling peptide interactions solely based on non-bonded interactions, as commonly done in an implicit-solvent like environment, does not necessarily guarantee improved affinity and selectivity. Instead, the contributions made by conformational entropies and solution penalties must also be carefully considered in the peptide design. While further work is certainly warranted before these *in silico* platforms could be reliably used to engineer peptides or direct peptide immobilization strategies for better biosensing efficacies, the mechanistic insights gained from the MD simulations can facilitate a more rational and guided approach to directing peptide modifications or determining site of immobilization or even in the selection of surface for a predictable ochratoxin sensing capabilities. In a broader sense, the methods outlined in the current study can be extended to virtually any peptide–hapten system, the adoption of MD approach could eventually fast-track the optimization of the sensing elements for a wide variety of haptens.

## 4. Materials and Methods

### 4.1. Simulation Protocol

Linear structure of the peptides was built from the primary sequence of the respective peptides using Visual Molecular Dynamics (version 1.9.8) [38]. Similarly, the initial coordinates and topology parameters for ochratoxins (OTA and OTB) were obtained from the ZINC database (ZINC12) and automated topology builder (ATB) and repository (2.1), respectively [39,40]. The amino acids and the hapten were modeled with the likely protonation state in a wine-like solution of pH 3–4. The N-termini, histidines, arginines, and lysines within the peptide were macrostateled as fully protonated. The C-termini, aspartic acid, glutamic acids, and ochratoxins were macrostateled as uncharged or neutral. 

A GROMOS 54A7 force field [41,42] and an SPC water macrostate were used to describe the molecular mechanics of the macrostate system. MD simulations were carried out in a GROMACS (version 5.0.4) simulator [43]. All simulations were done in a cubic box under periodic boundary conditions with the dimension of the simulation box chosen such that the minimum distance of the solutes to the box wall was at least 2.0 nm. During the initial stages of macrostate refinement, non-hydrogen atoms were restrained (1000 kcal/mol/Å^2^), and the system was relaxed by energy minimization using 10,000 steps of the steepest descent algorithm to remove steric clashes between atoms. The excess charges in the system were neutralized with 0.01 M sodium co-ion and chloride counter-ions. Long-range electrostatic interactions (real-space truncation at 1.4 nm and grid spacing of 0.12 nm) was handled using periodic boundary conditions with Particle Mesh Ewald (PME) summation, and was updated every 10 fs, together with the pair list. The Lennard–Jones 6–12 potential was used to evaluate the van der Waals interactions within a cutoff distance of 1.4 nm and was updated at every step. The LINCS algorithm was used to constrain the lengths of covalent bonds and the geometry of the water molecules. A coupling scheme using velocity rescaling with a stochastic term was applied to maintain the temperature at 298 K with the modified Berendsen coupling method and a relaxation time constant of 0.1 ps. Pressure of the system was maintained at 1 bar using the Parrinello–Rahman coupling method and a relaxation time constant of 2.0 ps. Initial velocities were generated randomly using Maxwell–Boltzmann distribution corresponding to 298 K. Neighbor lists were updated every 10 fs using a group cutoff scheme. The equilibrated peptide–hapten structures were obtained from the production runs of four regular MD initiated with different sets of random velocities sampled from a Maxwell–Boltzmann distribution. The production run of each replica involved 500 ns of simulation that were run under unrestrained isothermal–isobaric (NPT) ensemble at 298 K and 1 bar. Conformations were sampled every 10 ps.

### 4.2. Sampling of Kinetically Relevant Peptide and Peptide–Hapten Configurations

All BEMD simulations were performed on the NPT ensemble at 298 K and 1 bar using GROMACS (version 5.0.4) compiled with the PLUMED (version 2.2.2) plugin [44]. Prior to binding simulations, peptide configurations were sampled using the BEMD simulation [25,45]. A regular 100 ns MD simulation was initialized from the linear structure of the peptide. Three independent BEMD simulations were initialized from random configurations of the peptide. Each of the peptide configurations was sampled from regular MD simulation with an RMSD >2 nm. For each BEMD simulation of peptide folding, three replicas were used. Each of the first two replicas were biased by a different CV, namely, main-chain radius of gyration (R_g_) and number of main-chain hydrogen bonds within the peptide (N_hb_). The third replica was simulated without any bias. From the peptide folding simulations, four macrostates were identified and were used in the binding simulation. For each of the sampled peptide configurations, binding simulation was initiated by biasing five different CVs. The first four replicas was biased by a different CV, namely, main-chain radius of gyration (R_g_), number of main-chain hydrogen bonds between the peptide and haptens (N_hb_), degree of similarity between the torsional angles transversed by the peptide and hapten (Φ_corr_), and distance between the center of the hapten and center of the peptide (d_1_). A fifth replica without any bias was also simulated. Table 4 provides the parameters that were defined for each CV. Table 4 below outlines the parameters used in the current study. The selection of the parameters for different CVs were based on the guidelines outlined in a previous study [25]. Briefly, the bias potential was set as 0 beyond the interval range in Table 4. Gaussian potentials with a height of 0.2 kJ/mol were added at every 1 ps and exchange attempts between replicas was attempted every 200 ps.

The convergence and equilibrium state of the BEMD trajectories were verified by calculating the potential of mean force (PMF) along different CVs. The evolution of each replica in the free energy landscape was further monitored and then sequentially traced using the replica index. For the peptide folding and binding simulations, all CVs could reconstruct similar PMF profile. However, there were differences in the convergence time. In general, the CVs, R_g_, N_hb_, and d_1_, were able to reach convergence in relatively similar simulation time (<30 ns). PMF took longer to converge (>40 ns) when biased along Φ_cor_. When all 4 CVs were used, the average replica exchange probability was ∼0.26. After equilibration and convergence, the simulations were extended for an additional 100 ns. To avoid structural bias, four BEMD runs were initialized with four peptide conformations that were identified in Figure 3.

### 4.3. Predictive Modeling of the Peptide Folding and Binding Kinetics

MSM construction, analysis and validation were achieved using the functionalities provided with pyEMMA (version 2.2.3) [29,33]. Only the unbiased replicas from each BEMD simulation were used for MSM modeling.

#### 4.3.1. MSM Construction and Iteration

The first step towards estimating an MSM was to transform the simulation trajectories in the Cartesian coordinate space into trajectories in a preselected feature space that can capture the peptide conformational shifts. To fully resolve the conformational space of a peptide with N_atom_, it was required to analyze 3N_atom_ coordinates. For the binding simulations, the distance between the heavy atoms of the hapten and the peptide was used as the metric to resolve the conformational space. In our simulations, two atoms were considered to be in contact if their distance was less than 5 Å. Time independent component analysis (TICA) was performed on the featurized trajectories to find the slow linear sub-space of the input features. TICA components that account for 90% of the total kinetic variance in the configurations were retained for projection and analysis. For binding simulations, dimensionality reduction was achieved by projecting the TICA components onto five dimensions. Each TICA component was subsequently scaled according to its corresponding eigenvalue to obtain a kinetic map in which Euclidean distances was proportional to kinetic distances, providing an optimal space to perform clustering. Mini batch k-means clustering method was employed to group the snapshots into 100 microstates.

#### 4.3.2. Validation of MSM Macrostates

Markov macrostate was determined by analysis of the implied time scales, that is, the Markov macrostate’s relaxation timescales as a function of the lag time, ‘*τ*’. The parameter ‘*τ*’ is a crucial parameter that influences the accuracy of Markov macrostate. In our studies, we chose the lag time of 5 ns to build the MSM. The relaxation time scales did not change at longer lag times. The estimated Markov macrostate was validated using a Chapman-Kolmogorow test of the Markov macrostate [33]. The robustness of MSM was also assessed by increasing the number of clusters and projected dimensions [33].

#### 4.3.3. Estimation of Equilibrium Population Distribution and Binding Free Energy

Four macrostates were sampled by lumping kinetically close microstates using the Perron cluster cluster analysis (PCCA+) lumping algorithm. For each macrostate, the binding free energy (Equation (1)) and the equilibrium distribution of the peptides were computed from the reversible transition matrix by comparing the probabilities of bound to unbound microstates. The reversible transition matrix was estimated using the maximum likelihood estimator [33]:

(1) $$\Delta {G^{{}^{\circ}}}_{binding}\;=-\;kT\ln\left(\frac{\pi_{bound}}{\pi_{unbound}}\;\frac{V_{unbound}}{V_{0}}\right),$$

where *π_bound_* is the stationary probability of the bound state, *π_unbound_* is the stationary probability of the state including the cluster with zero peptide–hapten contacts, *V_unbound_* is the simulated solvent volume, and V_0_ = 1.663 nm^3^ is the standard volume.

The binding affinities and equilibrium population distribution of the peptides were derived from the MSM macrostate constructed using a lag time of 5 ns. Error estimates were calculated by 100 bootstrapping iterations, from each of which 5% of the trajectories were randomly eliminated.

### 4.4. Other Trajectory Analysis

Trajectories were visualized using Visual Molecular Dynamics (VMD version 1.9.8). Analysis tools provided within GROMACS (version 5.0.4) were used for estimating the RDF, root mean squared fluctuation (RMSF), the H-bonding profile, and for generating the contact maps [43]. DSSP analysis provided with MDtraj (version 1.7.2.) was used for secondary structure analysis [46]. Binding energy was decomposed as a cumulative sum of three main energetic components, namely, non-bonded energetic contributions, solvation penalty, and conformational entropy. The non-bonded energetic contributions and solvation penalties were estimated using g_mmpbsa, a GROMACS-based plugin for high-throughput MM/PBSA calculation [47]. As the plug-in do not currently support conformational entropy penalty calculations, these estimates were obtained by quasi-harmonic mode analysis, using the functionalities provided within GROMACS (version 5.0.4).

## Figures and Tables

**Table 1:** Structural and thermodynamic characteristics of the folded peptides.

Peptide Name	Num. of Residues ^1^	Preferred Macrostate ^2^	Basin Depth ^3^	2° Structure Preference ^4^	Pr.-Pr. H-Bonds ^5^	Pr.-Solv. H-Bonds ^6^
Hexamer(SNLHPK)	6	Model #3	2.7 kT	Random/Coil	2	24
Octamer (CSIVEDGK)	8	Model #4	4.9 kT	Random/Coil	8	20
NFO4 (VYMNRKYYKCCK)	12	Model #1	2.9 kT	Random/Coil	2	44
13-mer (GPAGIDGPAGIRC)	13	Model #3	5.0 kT	Random/Coil	6	18

^1^ Num. of Residues refers to the total number of amino acids constituting the peptide; ^2^ Preferred macrostate refers to the peptide configuration that was most populated at equilibrium; ^3^ Basin depths refer to the well depth corresponding to the preferred macrostate; ^4^ 2° structure preference refers to the overall structural preference of the preferred macrostate; ^5^ Pr-Pr. H-bonds refer to the average number of inter-peptide hydrogen bonds (h-bonds) in the preferred macrostate; ^6^ Pr-Solv. H-bonds refer to the average number of h-bonds between the peptide and water in the preferred macrostate.

**Table 2:** Structural and thermodynamic characteristics of the peptide–hapten complex.

System Name	Complex Dist. ^1^ (%)	Pref. State ^2^	Well Depth ^3^	Str. Shifts ^4^	Cont. Freq. ^5^	RMSF Shift ^6^	ΔG° (kJ/mol) ^7^
Hexamer-OTA	50.2	#3 (47%)	<3.0 kT	No	2/6	Decreased	−15.43 (1.92)
Hexamer-OTB	49.8	#3 (45%)	<3.0 kT	No	5/6	No Shift	−15.31 (1.42)
Octamer-OTA	52.0	#3 (44%)	~4.5 kT	No	3/8	Increased	−15.61 (1.92)
Octamer-OTB	49.6	#3 (54%)	~3.0 kT	No	8/8	Decreased	−14.52 (1.26)
NFO4-OTA	67.4	#3 (48%)	>7.0 kT	Yes	2/12	Increased	−33.34 (3.43)
NFO4-OTB	61.9	#1 (40%)	>7.0 kT	Yes	1/12	Increased	−26.99 (3.22)
13mer-OTA	56.7	#3 (37%)	<3.0 kT	No	5/13	Decreased	−14.81 (1.46)
13mer-OTB	50.6	#3 (48%)	<3.0 kT	No	2/13	Decreased	−13.64 (2.59)

^1^ Complex Dist. refers to the overall bound fraction (%) of the peptide–hapten complex; ^2^ Prefer. State refers to the most populated macrostate that corresponds to the bound state. Within the parenthesis, the percent contribution of the Prefer. State within the Complex Dist. s provided; ^3^ Well Depth refers to the energy barrier for the Prefer. State; ^4^ Str. Shifts refers to the 2*°* structure shifts in the preferred macrostate relative to the unbound state; ^5^ Cont. Freq. refers to the average number of residues within 0.35 nm of the hapten; ^6^ RMSF Shift refers to the structural fluctuation in the preferred macrostate relative to the unbound state; ^7^ Free energy of binding refers to the equilibrium binding free energy.

**Table 3:** Standard state binding free energies (mean ±95% C.I.) of synthetic peptides to ochratoxins in a wine-like solution pH.

Recognition Molecule	ΔG°_OTA-PRE_ (kJ/mol)	ΔG°_OTB-PRE_ (kJ/mol)	K_D_-_OTA_(μM) Pred. ^1^	Selectivity Pred. ^1^	K_D_-_OTA_(μM)-Expt
Albumin	-	-	-	-	0.019–1 ^2^
Antibody	-	-	-	-	0.00001–0.083 ^3^
DNA Aptamer	-	-	-	-	0.096–0.370 ^4^
Hexamer	−15.43 (1.92)	−15.31 (1.42)	1991	~1	29.4 ^5^
Octamer	−15.61 (1.42)	−14.52 (1.26)	1861	~2	11.8 ^6^
NFO4	−33.34 (3.43)	−26.99 (3.22)	1.47	~13	0.079 ^7^
13-mer	−14.81 (1.46)	−13.64 (2.59)	2563	~2	15.7 ^6^

^1^ The dissociation constant (K_D_) for ochratoxins was estimated from the average ΔG^°^ estimate using the Equation $K_{D}\;=\;\frac{1}{\exp\left(\;-\;\frac{\Delta G}{RT}\right)}$ where R = 0.00831 kJ mol^−1^ K^−1^and T = 298 K. Selectivity-Pred was based on the relative ratios of K_D-OTB_ to K_D-OTA_; ^2^ See Refs [1,2,14]. Not considered to be selective; ^3^ See Refs [7,16,34]. Estimate from immobilized system. 20 fold selective to OTA when immobilized; ^4^ See Refs [10,11,35,36]. Solution-based estimate. 6-100 fold selectivity to OTA; ^5^ See Refs [13,17]. Estimates based on standard solid-phase assay (K_D_ = 29.4 μM). Immobilization with non-standard solid substrate altered the original estimate (K_D_ ~ 0.01 μM–0.1 μM). No known data on the OTA selectivity; ^6^ See [14]’s estimates using SPR. No known data on the OTA selectivity; ^7^ See Ref [15] Estimate using HPLC-FLD. 3-fold selective to OTA when immobilized.

**Table 4:** Parameters used for BEMD simulations of peptides and peptide–hapten interactions.

Parameter	R_g_	N_hb_	Φ_corr_	d_1_
Sigma (kJ/mol)	0.020	0.500	0.400	0.025
Interval range	0.50–1.20	4–40	5–25	0.10–0.50
Bias factor	8	8	8	8
Deposition Frequency (ps)	1	1	1	1

## Supplementary Materials

The following supplementary materials are available online at www.mdpi.com/2072-6651/9/5/164/s1, Figure S1: Potential of mean force (PMF) curve for (a) Hexamer; (b) octamer; (c) NFO4 and (d) 13-mer. Figure S2: Radial distribution (RDF) of water around the folded peptide. Figure S3: Contact Map of the folded peptides, Figure S4: Average h-bond profile in folded peptides. Figure S5: Secondary structure preference of the folded peptides. Figure S6: RDF of water around the peptide–ochratoxin bound complexes. Figure S7: RMSF of the peptides in the peptide–ochratoxin bound complex. Figures S8 and S9: Secondary structural preference of the peptide in bound complex. Figures S10 and S11: Contact map of the residues within the peptide–hapten complex. Figures S12 and S13: H-bond profile within the bound complexes. Tables S1 and S2: Breakdown of the energetic components contributing to the overall binding energetics. Tables S3–S6: Residue-level contribution the overall binding energetics of each peptide system.
