# Supplementary material for: Evaluation of Ochratoxin Recognition by Peptides Using Explicit Solvent Molecular Dynamics
Source: Toxins (Basel). 2017 May 13;9(5):164. doi: 10.3390/toxins9050164 (PMC5450712; doi:10.3390/toxins9050164)
Supplement: Supplementary file 1 [file toxins-09-00164-s001.pdf]

Supplementary Materials: Evaluation of Ochratoxin Recognition by Peptides using Explicit Solvent Molecular Dynamics

Aby A. Thyparambil, Ingrid Bazin and Anthony Guiseppi-Elie

1. Peptide Folding Simulation

1.1. Convergence of the BEMD Simulations

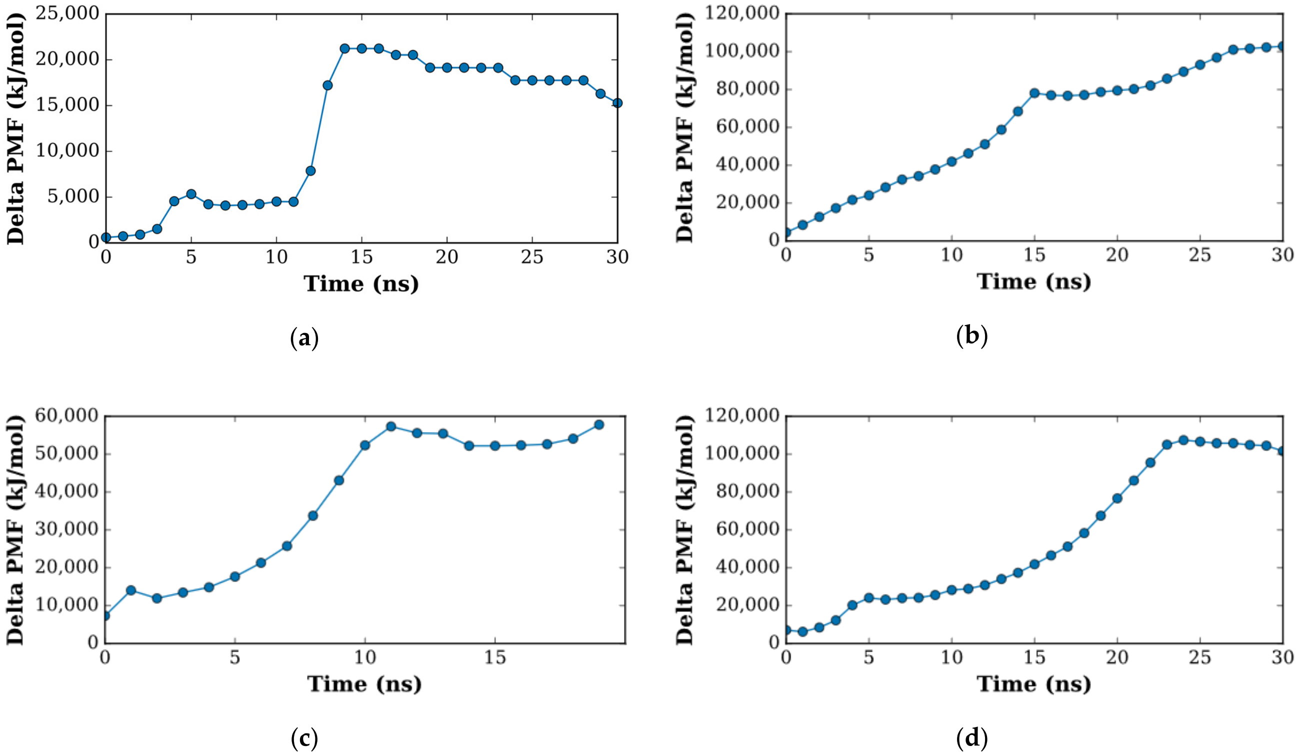

Figure S1. Convergence of potential of mean force (PMF) in (**a**) hexamer, (**b**) octamer, (**c**) NFO4, and (**d**) 13-mer systems. PMF was estimated along Rg and backbone RMSD.

1.2. Radial Distribution of Water Around the Folded Peptide

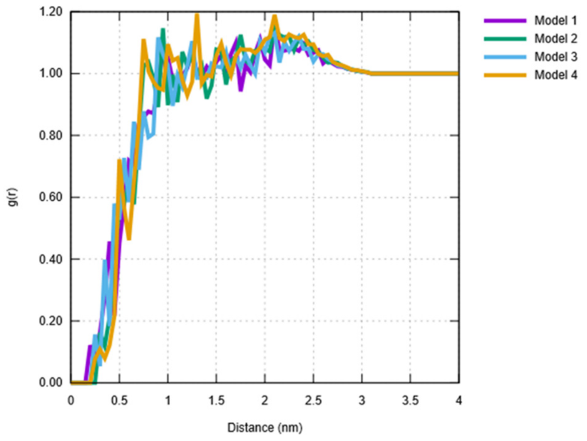

(**a**)

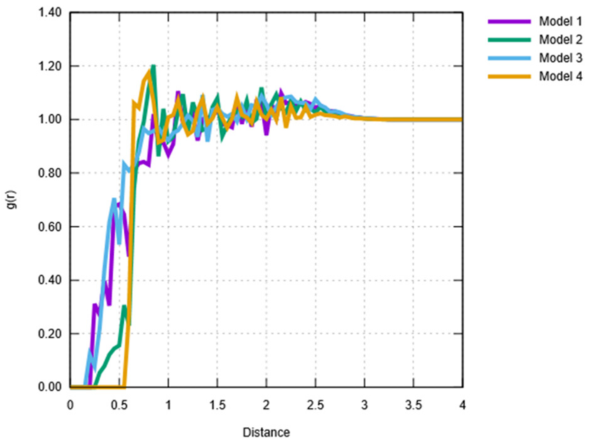

(**b**)

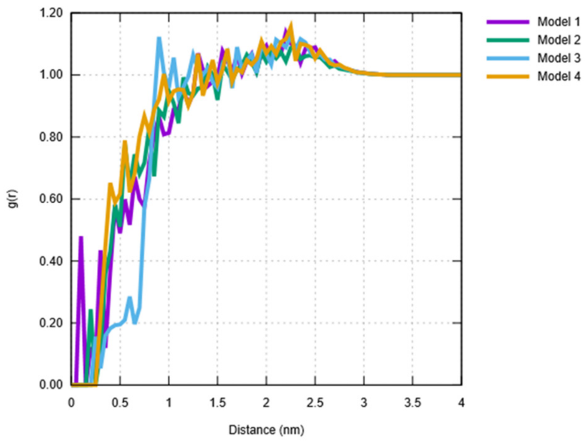

(**c**)

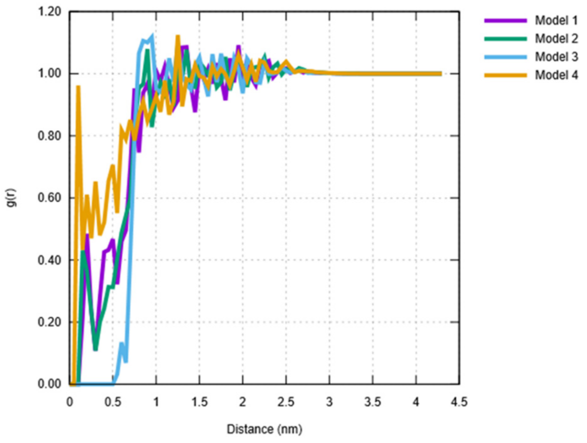

(**d**)

Figure S2. Radial distribution (RDF) of water around the top four lowest energy conformations of (**a**) hexamer (SNLHPK), (**b**) octamer (CSIVEDGK), (**c**) NFO4 (VYMNRKYYKCCK), and (**d**) 13-mer (GPAGIDGPAGIRC) identified from an independent component (IC) analysis of an accelerated sampling simulations. Model 1 is represented in violet, Model 2 is represented in green, Model 3 is represented in cyan, and Model 4 is represented in orange. RDF represents the density of water as a function of the distance from the peptide's center of mass (COM). The estimates are based on the mean of 50 frames of the respective models.

1.3. Contact Maps of the Folded Peptides

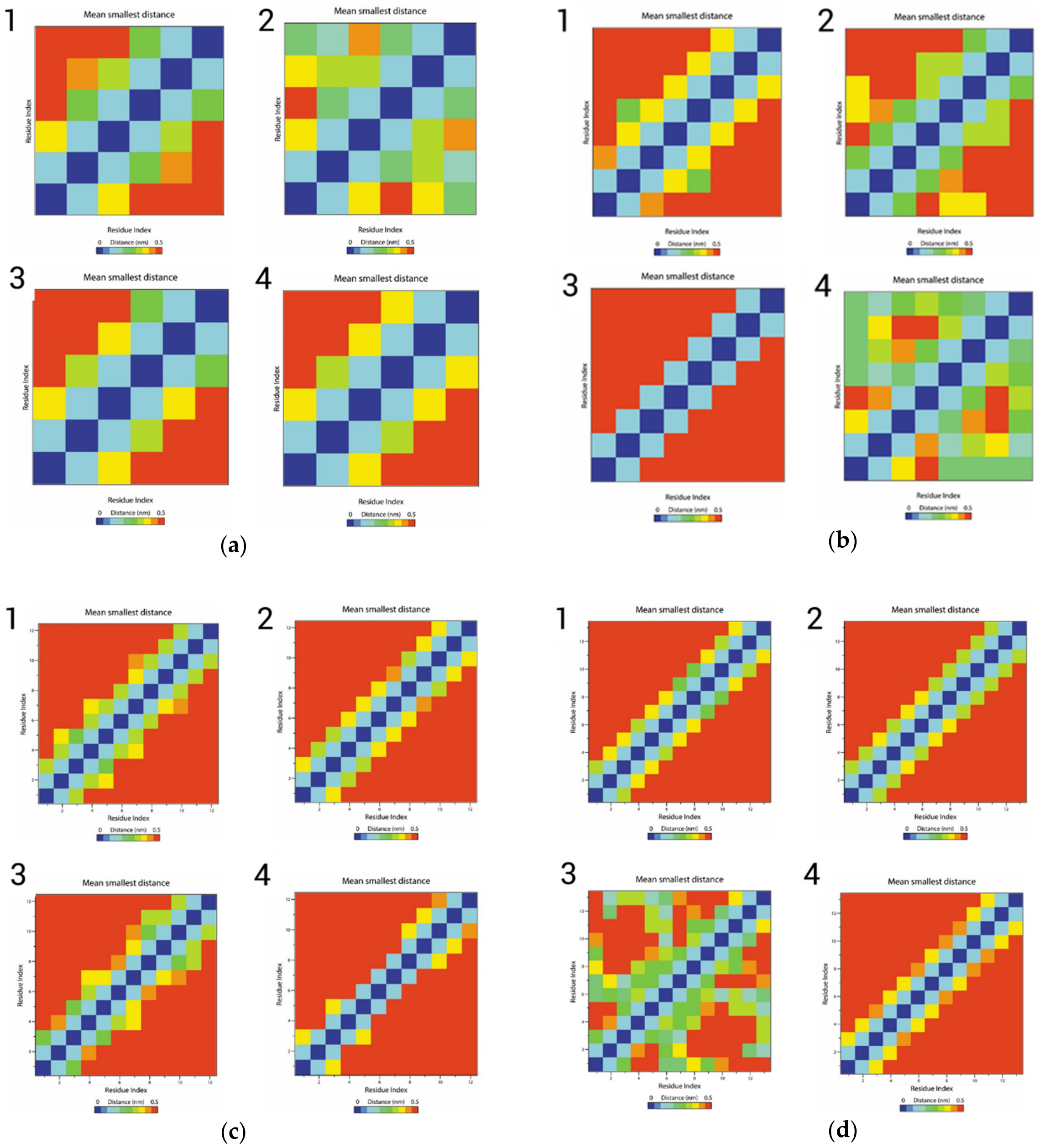

Figure S3. Contact maps of the top four lowest energy conformations of (**a**) hexamer (SNLHPK), (**b**) octamer (CSIVEDGK), (**c**) NFO4 (VYMNRKYYKCCK), and (**d**) 13-mer (GPAGIDGPAGIRC) identified from IC analysis of an accelerated sampling simulations. Each panel contains four sub-panels with '1' representing Model 1, '2' representing Model 2, '3' representing Model 3, and '4' representing Model 4. A contact map is a square map indicating the distance matrix between the amino acids constituting the peptide. The *x*- and *y*- axis of each contact map represent the residue position in the peptide. The color bar represents the distances from the respective *x*- and *y*- component, with colors nearer to the blue end of the scale bar representing shorter distances and colors nearer to the red end of the scale bar representing longer distances. Distances above 0.5 nm are represented in red. Transition of color occurs at every 0.05 nm. The diagonal matrix indicates the distance between the self-elements, which should be 0. The estimates are based on the mean of 50 frames of the respective models.

1.4. Average H-Bond Profile in Folded Peptides

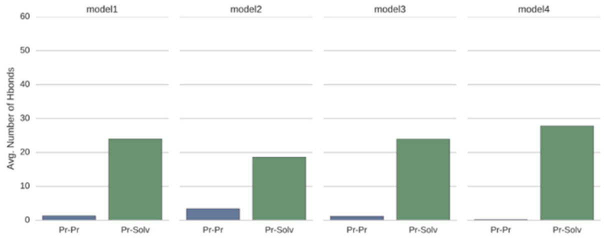

(**a**)

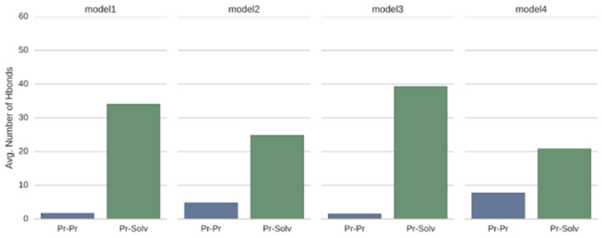

(**b**)

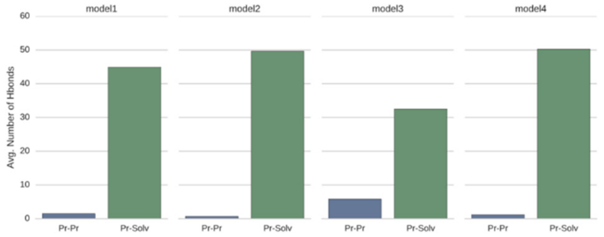

(**c**)

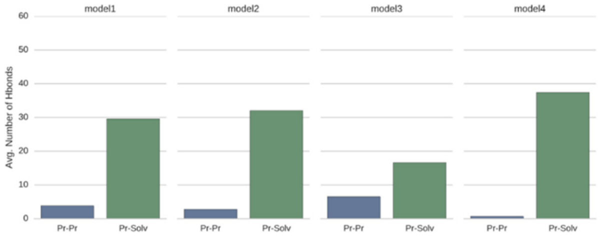

(**d**)

Figure S4. The average number of h-bonds within the top four lowest energy conformations of (**a**) hexamer (SNLHPK), (**b**) octamer (CSIVEDGK), (**c**) NFO4 (VYMNRKYYKCCK), and (**d**) 13-mer (GPAGIDGPAGIRC) identified from IC analysis of an accelerated sampling simulations. The blue bars represent the inter-molecular h-bonds within the peptide segments. The green bars indicate the H-bonds between the solvent and the peptides. The estimates are based on the mean of 50 frames of the respective models.

1.5. Secondary Structural Preference of the Folded Peptides

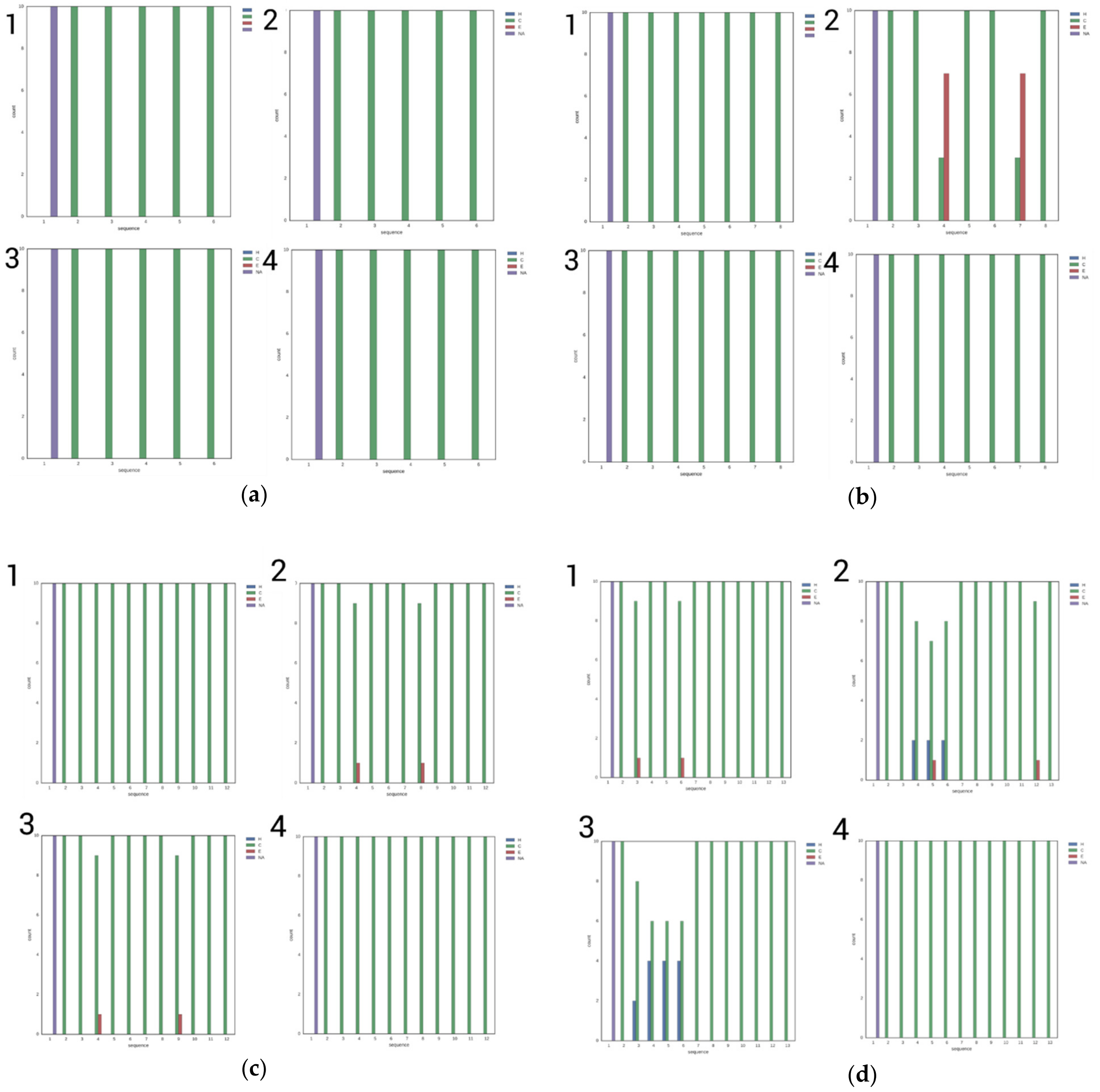

Figure S5. Secondary structure preference of each residues within the top four lowest energy conformations of (**a**) hexamer (SNLHPK), (**b**) octamer (CSIVEDGK), (**c**) NFO4 (VYMNRKYYKCCK), and (**d**) 13-mer (GPAGIDGPAGIRC) that were identified from IC analysis of an accelerated sampling simulations. Each panel contains four sub-panels, with '1' representing Model 1, '2' representing Model 2, '3' representing Model 3, and '4' representing Model 4. The *x*- axis represents the amino acid residue position within the peptide. The *y*-axis represents the average number of secondary structural elements. The color codes for the secondary structural elements are: helix (blue), sheets (red), coil/random loop (green). The estimates are based on the mean of 50 frames of the respective models.

2. Peptide–Hapten Binding Simulations.

2.1. Radial Distribution of Water Around the Bound Complex

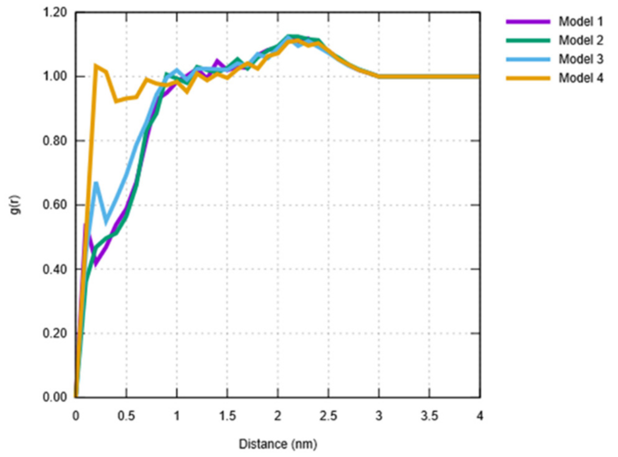

(**a**)

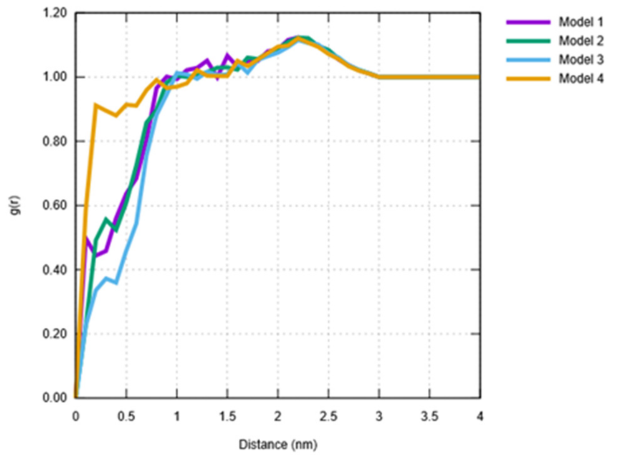

(**b**)

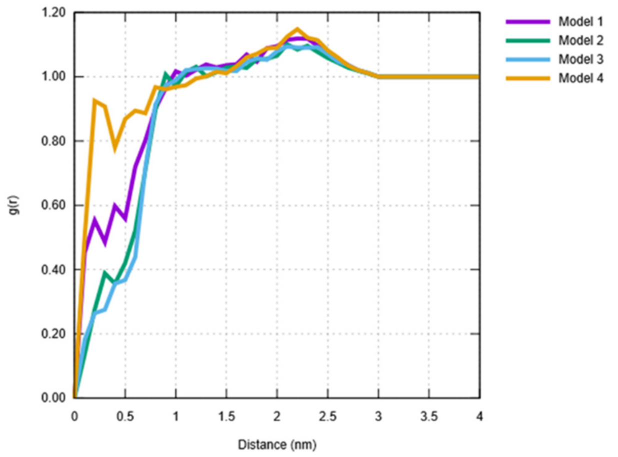

(**c**)

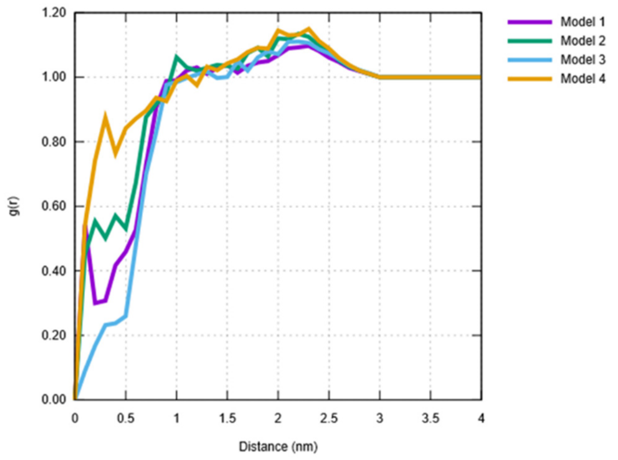

(**d**)

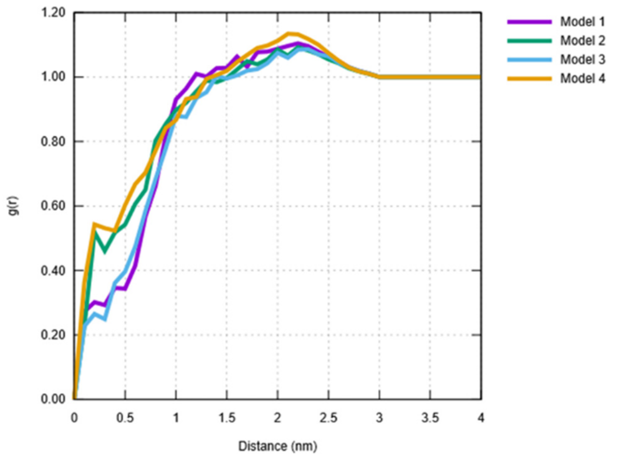

(**e**)

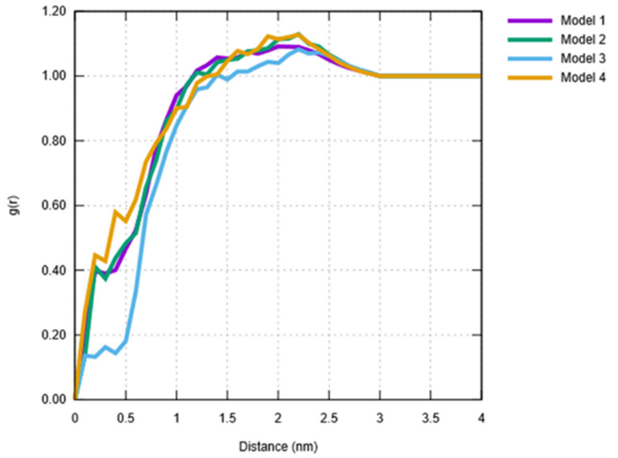

(**f**)

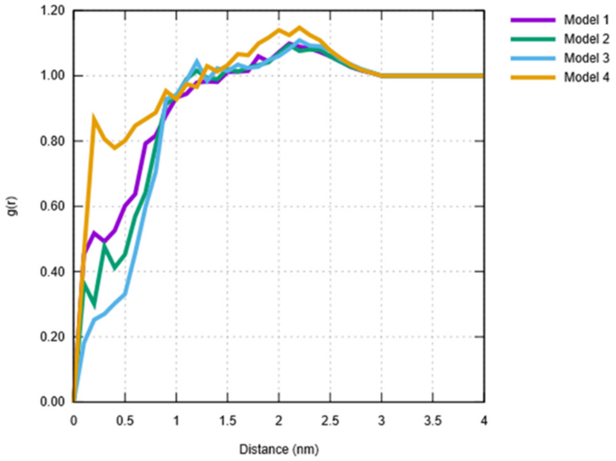

(**g**)

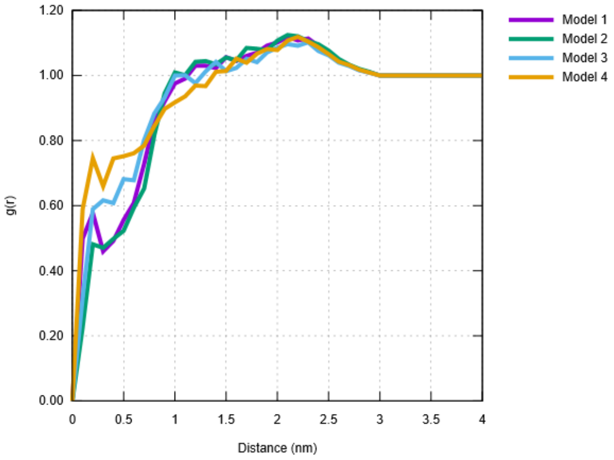

(**h**)

Figure S6. RDF of water around the top four lowest energy configurations of (**a**,**b**) hexamer (SNLHPK), (**c**,**d**) octamer (CSIVEDGK), (**e**,**f**) NFO4 (VYMNRKYYKCCK), and (**g**,**h**) 13-mer (GPAGIDGPAGIRC) when bound to (**a**,**c**,**e**,**g**) OTA and (**b**,**d**,**f**,**h**) OTB. RDF represents the density of water as a function of the distance from the complex's center of mass (COM). The estimates are based on the mean of 50 frames of the respective models.

2.2. Root Mean Squared Fluctuation (RMSF) of the Peptides in Bound Complex

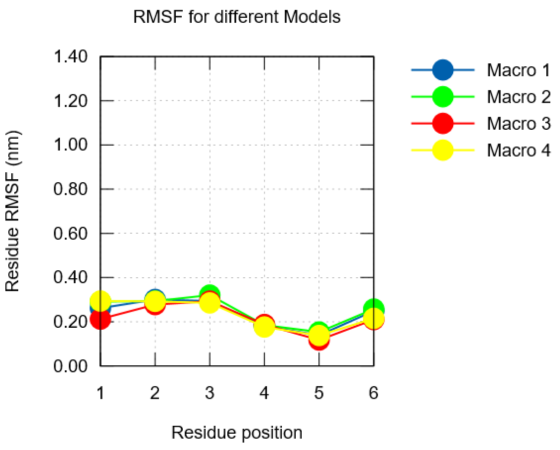

(**a**)

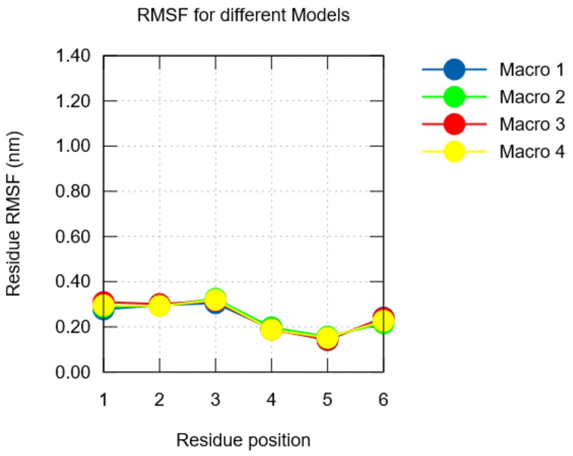

(**b**)

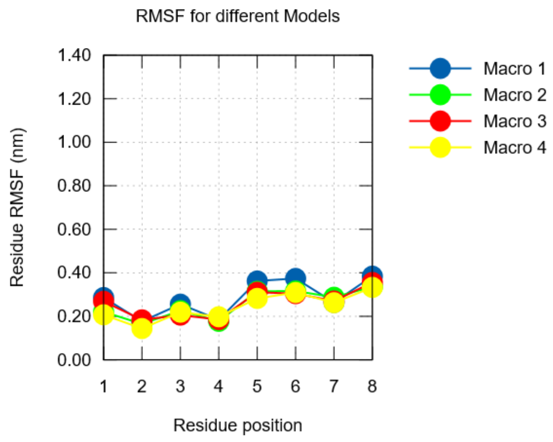

(**c**)

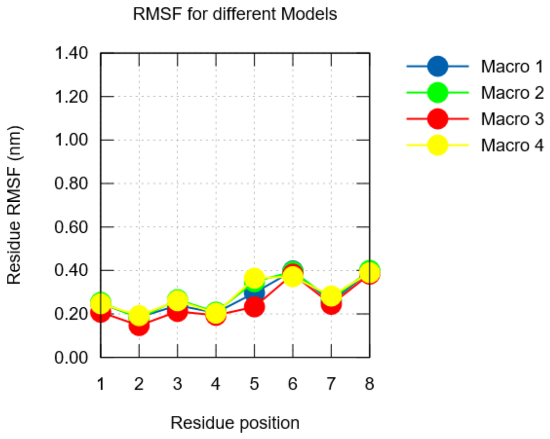

(**d**)

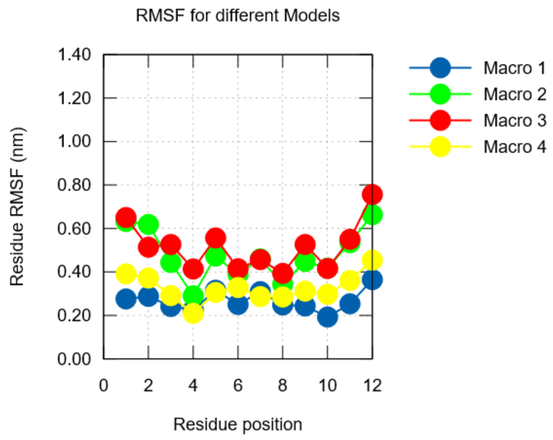

(**e**)

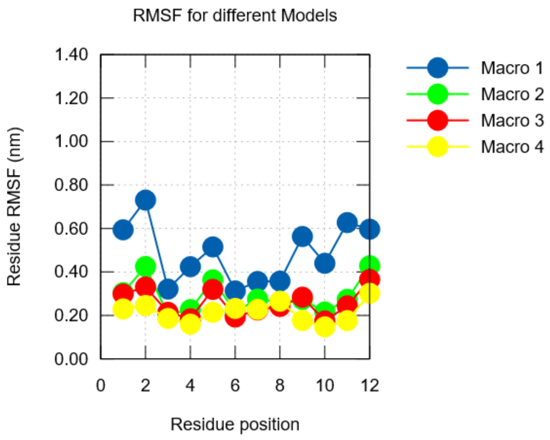

(**f**)

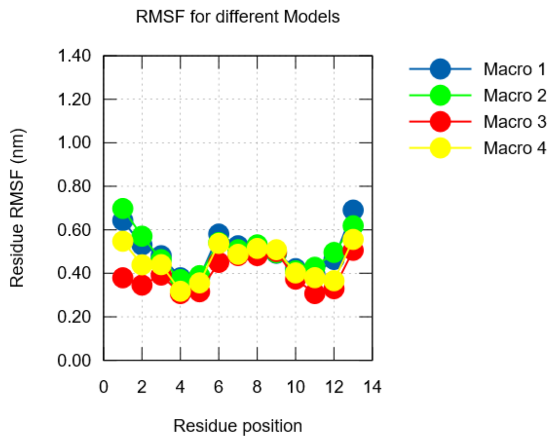

(**g**)

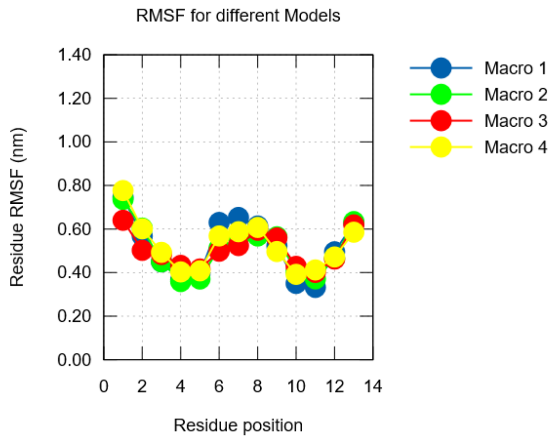

(**h**)

Figure S7. RMSF of the residue in the top four lowest energy configurations of (**a**,**b**) hexamer (SNLHPK), (**c**,**d**) octamer (CSIVEDGK), (**e**,**f**) NFO4 (VYMNRKYYKCCK) when bound to (**a**,**c**,**e**,**g**) OTA and (**b**,**d**,**f**,**h**) OTB. RDF represents the density of water as a function of the distance from the complex's center of mass (COM). The estimates are based on the mean of 50 frames of the respective models.

2.1.Secondary Structural Preference of Peptide when Interacting with Ochratoxins

2.1.1. OTA

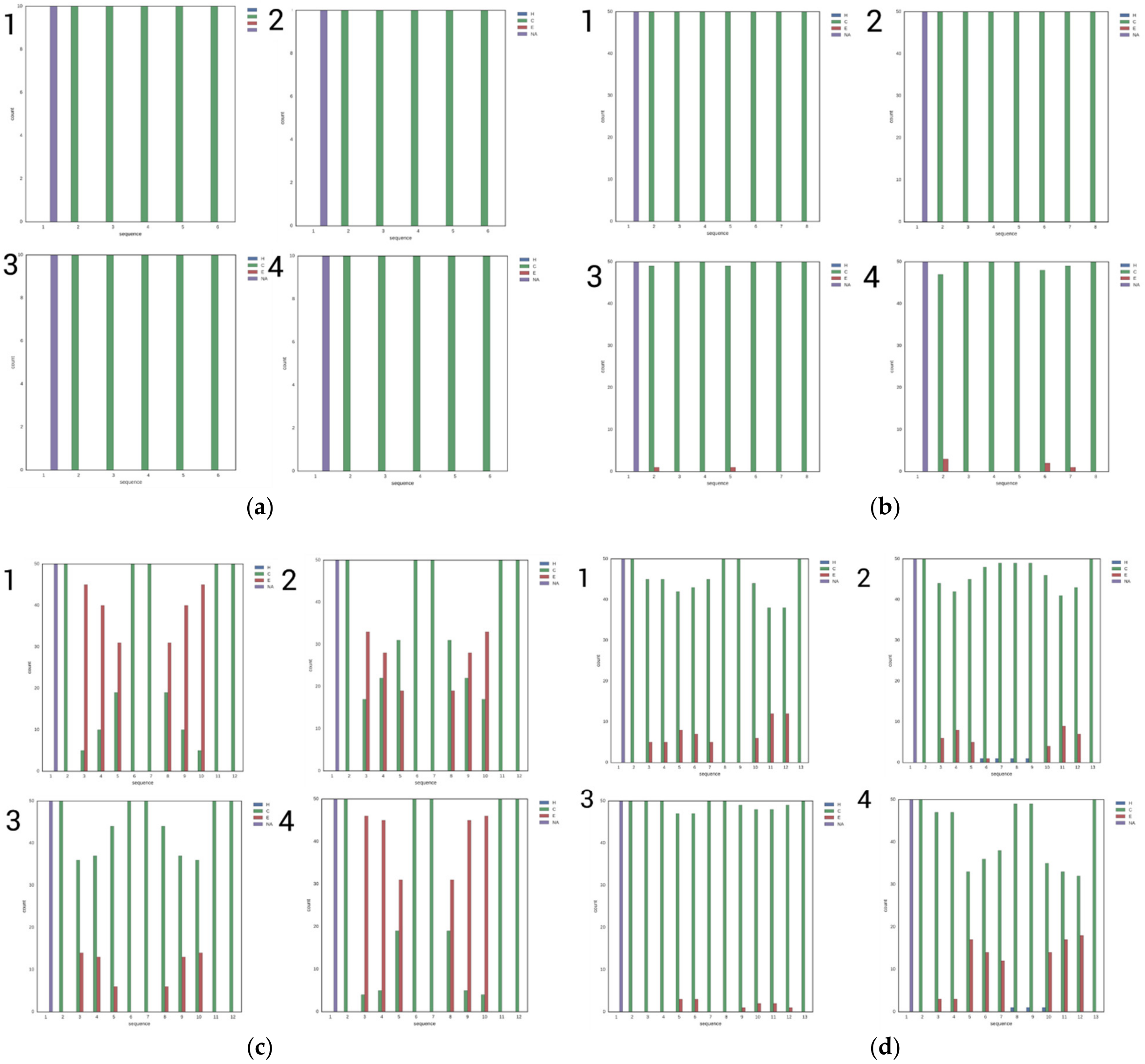

Figure S8. Secondary structure preference of each residues within the top four lowest energy conformations of (**a**) hexamer (SNLHPK), (**b**) octamer (CSIVEDGK), (**c**) NFO4 (VYMNRKYYKCCK), and (**d**) 13-mer (GPAGIDGPAGIRC) when complexed with OTA. Each panel contains four sub-panels with '1' representing Model 1, '2' representing Model 2, '3' representing Model 3, and '4' representing Model 4. The *x*- axis represents the amino acid residue position within the peptide. The *y*-axis represents the average number of secondary structural elements. The color codes for the secondary structural elements are: helix (blue), sheets (red), coil/random loop (green). The estimates are based on the mean of 50 frames representing the individual model.

2.1.2. OTB

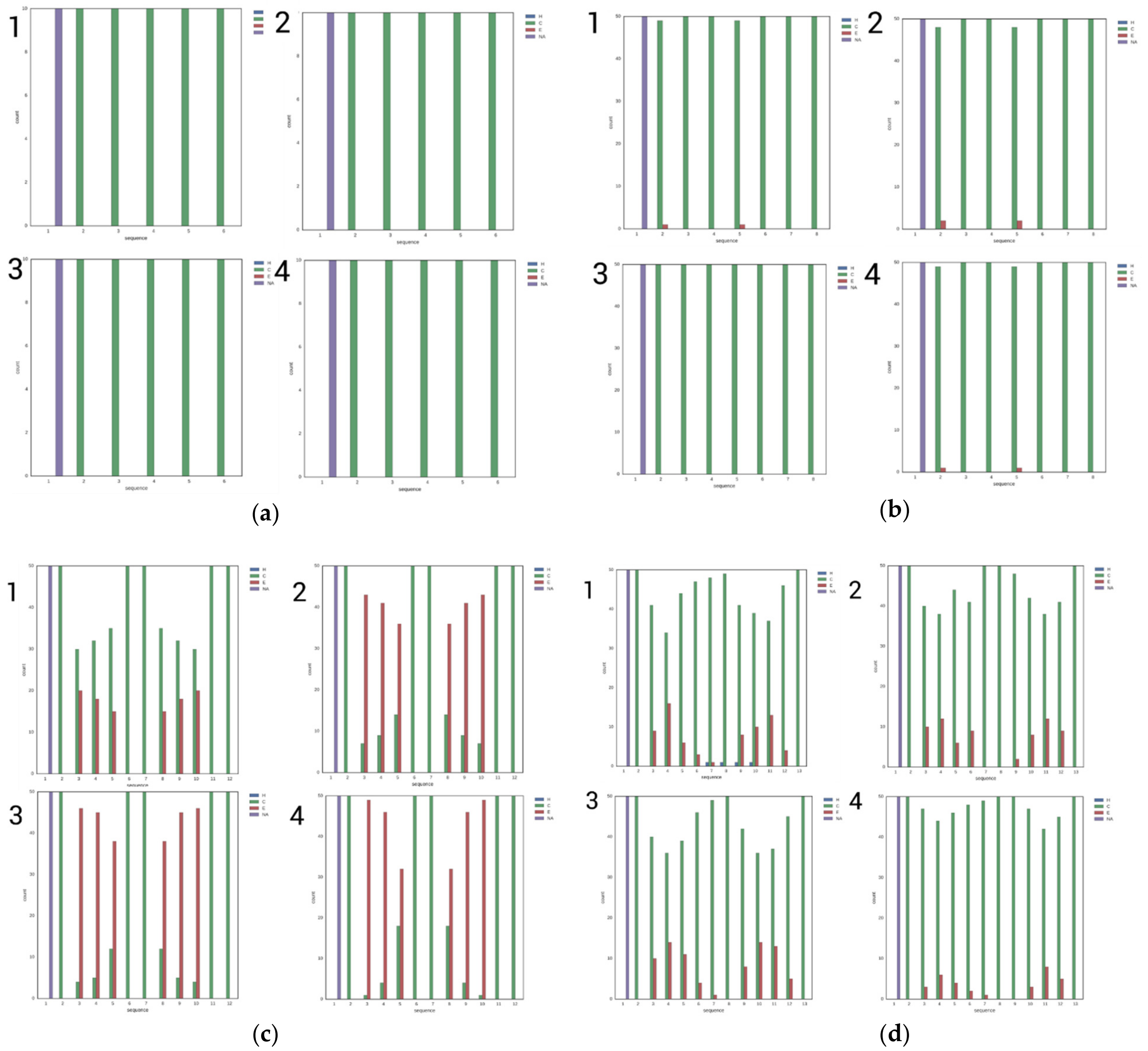

Figure S9. Secondary structure preference of each residues within the top four lowest energy conformations of (**a**) hexamer (SNLHPK), (**b**) octamer (CSIVEDGK), (**c**) NFO4 (VYMNRKYYKCCK), and (**d**) 13-mer (GPAGIDGPAGIRC) when complexed with OTB. Each panel contains four sub-panels, with '1' representing Model 1, '2' representing Model 2, '3' representing Model 3, and '4' representing Model 4. The *x*- axis represents the amino acid residue position within the peptide. The *y*-axis represents the average number of secondary structural elements. The color codes for the secondary structural elements are: helix (blue), sheets (red), coil/random loop (green). The estimates are based on the mean of 50 frames representing the individual model.

2.2. *Contact Maps of Peptides when Interacting with Ochratoxins*

2.2.1. OTA

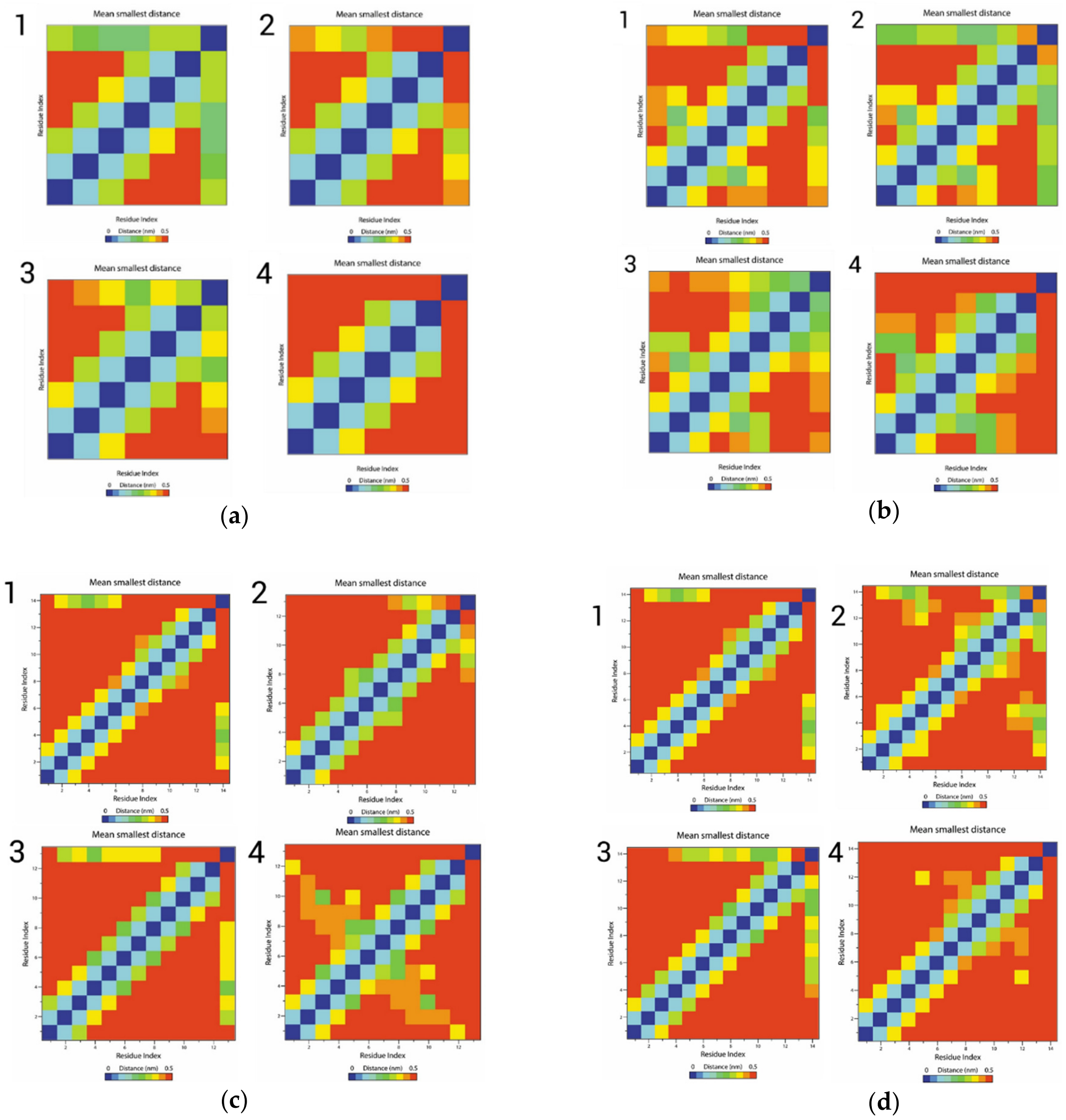

Figure S10. Contact maps of the top four lowest energy conformations of (**a**) hexamer (SNLHPK), (**b**) octamer (CSIVEDGK), (**c**) NFO4 (VYMNRKYYKCCK), and (**d**) 13-mer (GPAGIDGPAGIRC) when bound to OTA. Each panel contains four sub-panels with '1' representing Model 1, '2' representing Model 2, '3' representing Model 3, and '4' representing Model 4. A contact map is a square map indicating the distance matrix between the amino acids constituting the peptide and OTA. The *x*- and *y*- axis of each contact map represent the residue position in the peptide, with OTA as the last element. The color bar represents the distances from the respective *x*- and *y*-components. A color nearer to the blue end of the scale bar represents a shorter distance and colors nearer to the red end of the scale bar represent longer distances. Distances above 0.5 nm are represented in red. Transition of color occurs at every 0.05 nm. The diagonal matrix indicates the distance between the self-elements, which should be 0. The estimates are based on the mean of 50 frames representing the individual model.

2.2.2. OTB

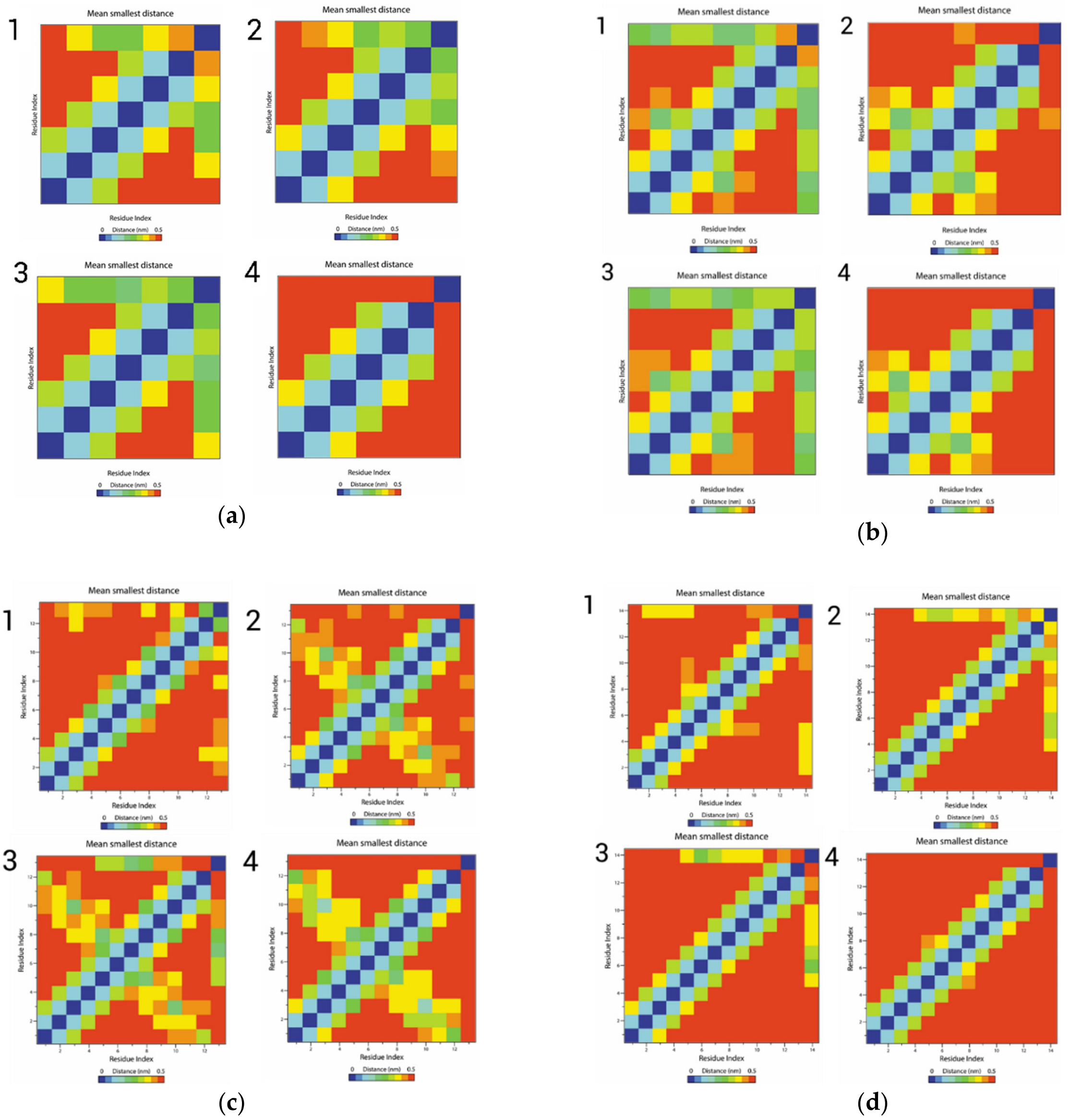

Figure S11. Contact maps of the top four lowest energy conformations of (**a**) hexamer (SNLHPK), (**b**) octamer (CSIVEDGK), (**c**) NFO4 (VYMNRKYYKCCK), and (**d**) 13-mer (GPAGIDGPAGIRC) when bound to OTB. Each panel contains four sub-panels, with '1' representing Model 1, '2' representing Model 2, '3' representing Model 3, and '4' representing Model 4. A contact map is a square map indicating the distance matrix between the amino acids constituting the peptide and OTB. The *x*- and *y*- axis of each contact map represent the residue position in the peptide, with OTB as the last element. The color bar represents the distances from the respective *x*- and *y*-components. A color nearer to the blue end of the scale bar represents a shorter distance and colors nearer to the red end of the scale bar represent longer distances. Distances above 0.5 nm are represented in red. Transition of color occurs at every 0.05 nm. The diagonal matrix indicates the distance between the self-elements, which should be zero. The estimates are based on the mean of 50 frames representing the individual model.

2.3. Histogram of H-bonding in Peptides when Interacting with Ochratoxins

2.3.1. OTA

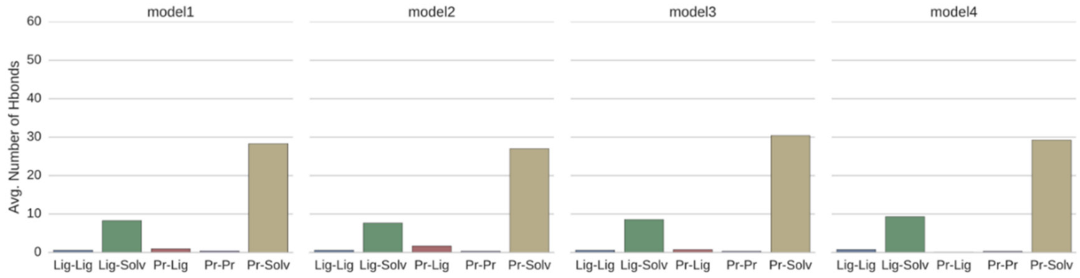

(**a**) Hexamer

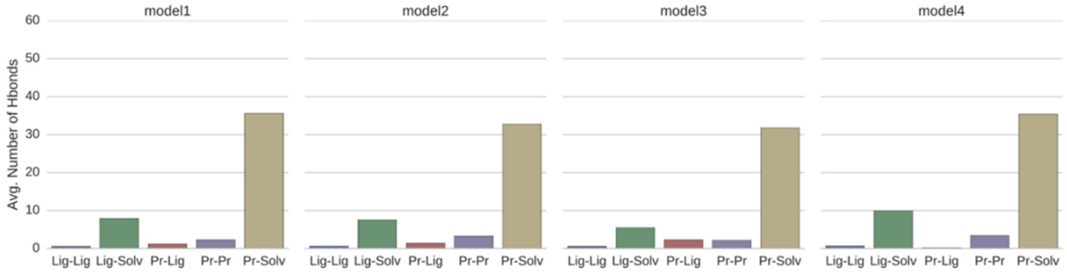

(**b**) Octamer

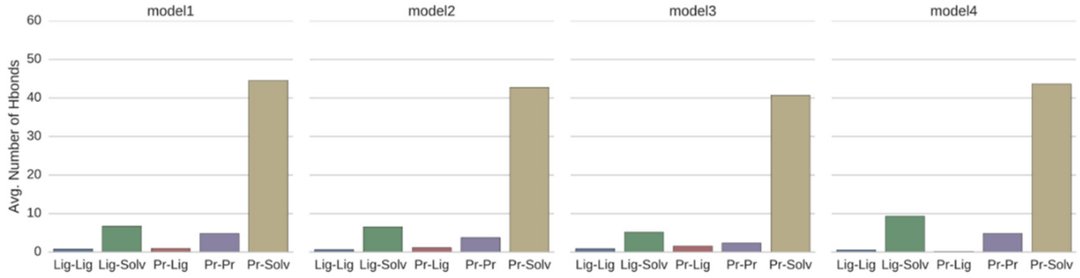

(**c**) NFO4

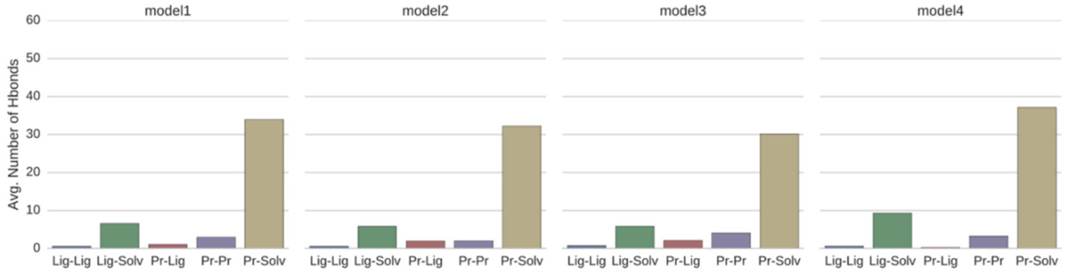

(**d**) 13-mer

Figure S12. The average number of h-bonds within the top four lowest energy conformations of (**a**) hexamer (SNLHPK), (**b**) octamer (CSIVEDGK), (**c**) NFO4 (VYMNRKYYKCCK), and (**d**) 13-mer (GPAGIDGPAGIRC) when bound to OTA. The blue bar represents the inter-molecular H-bonds within the hapten, the green bar represents the H-bonding between the hapten and solvent, the red bar represents the h-bonding between the peptide and the hapten, the purple bar represents the H-bonding within the peptide, and the brown bar represents the H-bonding between the peptide and the solvent. The estimates are based on the mean of 50 frames representing the individual model.

2.3.2. Frequency of H-Bond Solvent in OTB-Bound Peptide Complexes

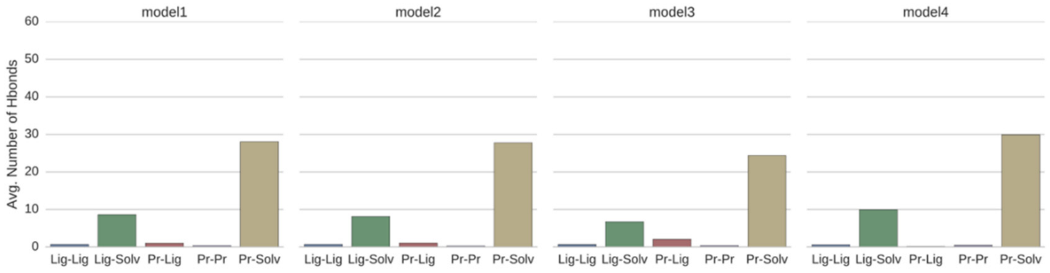

(**a**) Hexamer

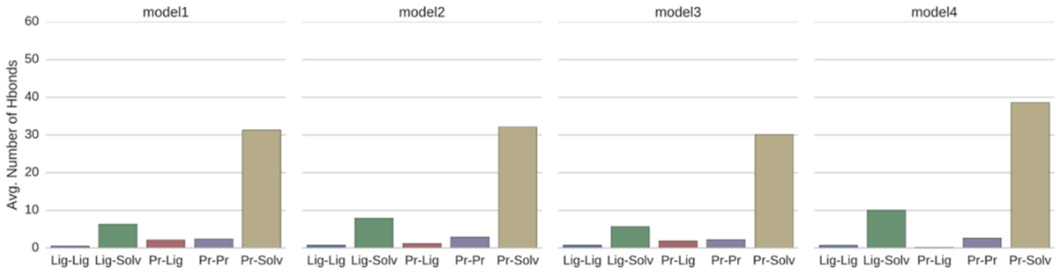

(**b**) Octamer

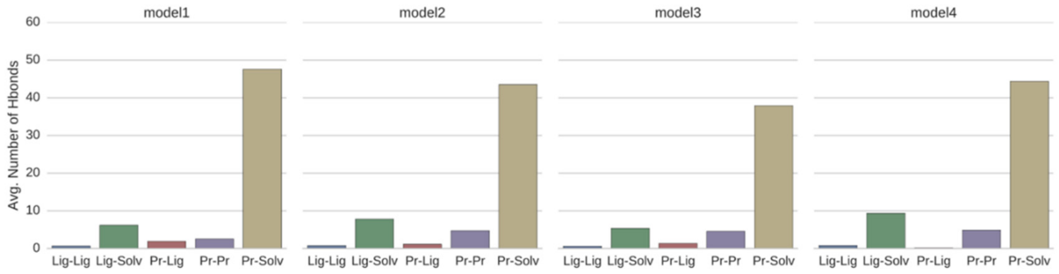

(**c**) NFO4

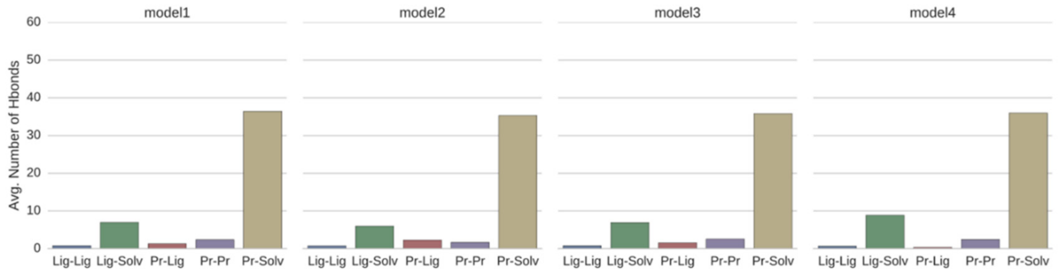

(**d**) 13-mer

Figure S13. The average number of H-bonds within the top four lowest energy conformations of (**a**) hexamer (SNLHPK), (**b**) octamer (CSIVEDGK), (**c**) NFO4 (VYMNRKYYKCCK), and (**d**) 13-mer (GPAGIDGPAGIRC) when bound to OTB. The blue bar represents the inter-molecular H-bonds within the hapten, the green bar represents the H-bonding between the hapten and the solvent, the red bar represents the H-bonding between the peptide and the hapten, the purple bar represents the H-bonding within the peptide, and the brown bar represents the H-bonding between the peptide and solvent. The estimates are based on the mean of 50 frames representing the individual model.

Table S1. Energetic components involved in peptide–OTB binding. Averaged from all models.

Energy Component	Hexamer	Octamer	NFO4	13-mer
van der Waals energy (kJ/mol)	–33.1 (6.3)	–52.8 (13.3)	–90.3 (22.9)	–66.5 (21.5)
Electrostatic energy (kJ/mol)	–21.3 (7.9)	–64.4 (16.6)	–34.3 (2.4)	–52.5 (13.8)
Non-bonded Interactions (kJ/mol)	–54.4 (13.5)	–117.2 (29.2)	–124.6 (23.1)	–119 (35.1)
Polar solvation energy (kJ/mol)	44.5 (12.8)	101.6 (25.9)	84.8 (8.0)	98.8 (25.7)
SASA energy (kJ/mol)	–4.5 (1.0)	–7.03 (1.6)	–10.4 (2.0)	–8.4 (2.2)
Solvation Penalty (kJ/mol)	40.0 (11.9)	94.6 (24.4)	74.4 (7.0)	90.4 (23.5)
Entropy-Bound (kJ $mol^{-1}K^{-1}$)	–0.120	–0.202	–0.327	–0.247
Entropy-Unbound (kJ $mol^{-1}K^{-1}$)	–0.117	–0.177	–0.282	–0.209
TΔS (kJ/mol)	–0.9	–7.5	–13.4	–11.3
Binding Energy (kJ/mol)	–13.5 (3.8)	–15.1 (8.1)	–36.8 (19.2)	–17.3 (8.9)

Table S2. Energetic components involved in peptide–OTB binding. Averaged from all models.

Energy Component	Hexamer	Octamer	NFO4	13-mer
van der Waals energy (kJ/mol)	–36.4 (10.0)	–58.5 (15.0)	–74.1 (18.4)	–55.1 (12.3)
Electrostatic energy (kJ/mol)	–15.7 (13.2)	–65.3 (18.8)	–47.5 (17.0)	–41.1 (10.8)
Non-bonded Interactions (kJ/mol)	–62.5 (21.6)	–123.8 (33.6)	–121.6 (35.1)	–96.2 (22.2)
Polar solvation energy (kJ/mol)	36.1 (24.2)	113.6 (34.7)	96.4 (35.6)	84.8 (23.7)
SASA energy (kJ/mol)	–4.9 (1.5)	–8.2 (2.0)	–9.5 (2.5)	–6.9 (1.6)
Solvation Penalty (kJ/mol)	31.2 (22.9)	105.4 (32.6)	86.9 (33.2)	77.9 (22.1)
Entropy-Bound (kJ $mol^{-1}K^{-1}$)	–0.126	–0.147	–0.283	–0.322
Entropy-Unbound (kJ $mol^{-1}K^{-1}$)	–0.124	–0.128	–0.245	–0.313
TΔS (kJ/mol)	–0.6	–5.7	–11.3	–2.7
Binding Energy (kJ/mol)	–13.6 (5.8)	–12.7 (4.2)	–23.4 (8.6)	–15.6 (2.7)

Table S3a. Residue-wise energy contribution for the hexamer (SNLHPK)–OTA (Macrostate 3) complex.

Residue	Non-Bonded (kJ/mol)	Polar (kJ/mol)	Non-Polar (kJ/mol)	Net Energy (kJ/mol)
SER-1	–0.7 (0.3)	0.3 (1.0)	–0.1 (0.1)	0.1 (0.5)
ASN-2	–2.1 (0.5)	0.5 (2.7)	–0.3 (0.1)	0.3 (0.4)
LEU-3	–4.5 (0.9)	0.9 (2.2)	–0.4 (0.1)	–2.8 (0.6)
HIS-4	–5.9 (1.2)	1.2 (4.9)	–0.4 (0.1)	–1.4 (0.4)
PRO-5	–2.2 (0.5)	0.5 (0.5)	–0.2 (0.1)	–1.9 (0.4)
LYS-6	–5.8 (1.4)	1.4 (9.9)	–0.4 (0.1)	3.7 (1.5)

Table S3b. Residue-wise energy contribution for the hexamer (SNLHPK)–OTB (Macrostate 3) complex.

Residue	Non-Bonded (kJ/mol)	Polar (kJ/mol)	Non-Polar (kJ/mol)	Net Energy (kJ/mol)
SER-1	–4.2 (1.0)	6.1 (1.6)	–0.4 (0.1)	1.5 (0.8)
ASN-2	–8.9 (1.2)	9.9 (1.3)	–0.7 (0.1)	0.4 (0.4)
LEU-3	–7.7 (0.9)	4.7 (0.7)	–0.6 (0.1)	–3.6 (0.5)
HIS-4	–7.3 (0.9)	5.1 (0.9)	–0.5 (0.1)	–2.7 (0.6)
PRO-5	–3.8 (0.5)	0.5 (0.2)	–0.3 (0.1)	–3.6 (0.5)
LYS-6	–10.3 (1.8)	20.2 (3.9)	–0.7 (0.1)	9.3 (2.2)

Table S4a. Residue-wise energy contribution for the octamer (CSIVEDGK)–OTA (Macrostate 3) complex.

Residue	Non-Bonded (kJ/mol)	Polar (kJ/mol)	Non-Polar (kJ/mol)	Net Energy (kJ/mol)
CYS-1	–12.8 (1.5)	22.8 (2.8)	–0.7 (0.1)	9.2 (1.6)
SER-2	–4.7 (0.7)	4.3 (0.9)	–0.2 (0.1)	–0.6 (0.5)
ILE-3	–6.3 (0.7)	1.0 (0.2)	–0.6 (0.1)	–5.9 (0.6)
VAL-4	–4.9 (0.5)	0.6 (0.1)	–0.4 (0.1)	–4.6 (0.5)
GLU-5	–5.4 (1.2)	3.6 (1.8)	–0.2 (0.1)	–2 (0.9)
ASP-6	–17.7 (2.2)	28.5 (3.7)	–0.6 (0.1)	10.1 (1.6)
GLY-7	–2.4 (0.4)	1.3 (0.3)	–0.2 (0.1)	–1.3 (0.2)
LYS-8	–4.3 (0.9)	7.7 (1.7)	–0.2 (0.1)	3.2 (1.1)

Table S4b. Residue-wise energy contribution for the octamer (CSIVEDGK)–OTB (Macrostate 3) complex.

Residue	Non-Bonded (kJ/mol)	Polar (kJ/mol)	Non-Polar (kJ/mol)	Net Energy (kJ/mol)
CYS-1	–14.8 (1.8)	24.2 (3.4)	–0.8 (0.1)	8.7 (2.0)
SER-2	–5.3 (0.7)	4.6 (0.8)	–0.3 (0.1)	–0.9 (0.4)
ILE-3	–4.7 (0.5)	0.7 (0.2)	–0.4 (0.1)	–4.4 (0.5)
VAL-4	–4.8 (0.4)	0.8 (0.3)	–0.5 (0.1)	–4.5 (0.4)
GLU-5	–8.3 (1.4)	8.2 (2.2)	–0.4 (0.1)	–0.7 (1.0)
ASP-6	–15.2 (1.5)	24.1 (3.3)	–0.7 (0.1)	8.2 (2.0)
GLY-7	–3.9 (0.6)	3 (0.6)	–0.4 (0.1)	–1.3 (0.3)
LYS-8	–4.9 (1.0)	10 (2.4)	–0.3 (0.1)	4.6 (1.6)

Table S5a. Residue-wise energy contribution for the NFO4 (VYMNRKYYKCCK)–OTA (Macrostate 3) complex.

Residue	Non-Bonded (kJ/mol)	Polar (kJ/mol)	Non-Polar (kJ/mol)	Net Energy (kJ/mol)
VAL-1	−3 (0.7)	2.6 (0.8)	−0.3 (0.1)	−0.7 (0.5)
TYR-2	−11.9 (1.3)	5.7 (0.7)	−1.1 (0.1)	−7.3 (1.0)
MET-3	−8.6 (1.0)	5.5 (0.7)	−0.5 (0.1)	−3.6 (0.6)
ASN-4	−8.4 (0.9)	8.3 (0.9)	−0.5 (0.1)	−0.7 (0.4)
ARG-5	−3.5 (0.7)	2.5 (1.0)	−0.2 (0.1)	−1.2 (0.5)
LYS-6	−8.3 (1.3)	8.7 (1.8)	−0.5 (0.1)	−0.1 (0.8)
TYR-7	−5.5 (0.9)	2.1 (0.5)	−0.5 (0.1)	−3.9 (0.7)
TYR-8	−5.5 (0.8)	3.5 (0.5)	−0.6 (0.1)	−2.5 (0.6)
LYS-9	−2.1 (0.6)	1.5 (1.1)	−0.1 (0.1)	−0.8 (0.6)
CYS-10	−3 (0.7)	2.2 (0.6)	−0.2 (0.1)	−1.0 (0.3)
CYS-11	−0.7 (0.3)	0.3 (0.3)	−0.1 (0.1)	−0.5 (0.2)
LYS-12	−1.6 (0.7)	4.2 (1.7)	−0.1 (0.1)	2.5 (1.1)

Table S5b. Residue-wise energy contribution for the NFO4 (VYMNRKYYKCCK)–OTB (Macrostate 1) complex.

Residue	Non-Bonded (kJ/mol)	Polar (kJ/mol)	Non-Polar (kJ/mol)	Net Energy (kJ/mol)
VAL-1	−1.9 (0.4)	−0.1 (0.5)	−0.3 (0.1)	−2.3 (0.5)
TYR-2	−2.2 (0.3)	2.8 (0.4)	−0.2 (0.1)	0.4 (0.2)
MET-3	−6.1 (0.7)	1 (0.6)	−0.6 (0.1)	−5.8 (0.7)
ASN-4	−5.6 (0.9)	7.7 (0.9)	−0.4 (0.1)	1.7 (0.4)
ARG-5	−5.9 (0.6)	6.8 (1.4)	−0.6 (0.1)	0.3 (0.4)
LYS-6	−3.5 (0.7)	−1 (0.5)	−0.3 (0.1)	−4.8 (0.7)
TYR-7	−5.9 (1.1)	2.9 (0.4)	−0.5 (0.1)	−3.5 (0.7)
TYR-8	−11 (0.4)	6.9 (0.8)	−1 (0.1)	−5.2 (0.6)
LYS-9	−1.6 (0.5)	0.8 (0.5)	−0.1 (0.1)	−0.8 (0.5)
CYS-10	−3.2 (0.3)	1 (0.4)	−0.4 (0.1)	−2.6 (0.4)
CYS-11	−0.7 (2.5)	0.4 (0.3)	−0.1 (0.1)	−0.3 (0.2)
LYS-12	−21.3 (6.8)	42.9 (4.7)	−0.6 (0.1)	21.1 (2.3)

Table S6a. Residue-wise energy contribution for the 13-mer (GPAGIDGPAGIRC)–OTA (Macrostate 3) complex.

Residue	Non-Bonded (kJ/mol)	Polar (kJ/mol)	Non-Polar (kJ/mol)	Net Energy (kJ/mol)
GLY-1	0.3 (0.6)	0.7 (1.1)	–0.1 (0.1)	1.0 (0.6)
PRO-2	–2.9 (0.5)	1.0 (0.3)	–0.4 (0.1)	–2.3 (0.4)
ALA-3	–5.6 (0.6)	5.8 (0.6)	–0.6 (0.1)	–0.4 (0.3)
GLY-4	–8.5 (0.9)	5.8 (0.7)	–0.6 (0.1)	–3.3 (0.4)
ILE-5	–4.4 (0.4)	0.4 (0.2)	–0.4 (0.1)	–4.5 (0.4)
ASP-6	–1.7 (0.6)	2.5 (0.9)	–0.1 (0.1)	0.7 (0.4)
GLY-7	–0.2 (0.1)	–0.4 (0.1)	0 (0.1)	–0.6 (0.2)
PRO-8	–0.4 (0.2)	–0.2 (0.2)	0 (0.1)	–0.6 (0.1)
ALA-9	–0.5 (0.2)	0 (0.2)	0 (0.1)	–0.6 (0.1)
GLY-10	–1.9 (0.4)	1.7 (0.5)	–0.3 (0.1)	–0.5 (0.3)
ILE-11	–3.9 (0.5)	1.5 (0.3)	–0.2 (0.1)	–2.6 (0.3)
ARG-12	–21.6 (2.0)	27.5 (2.7)	–1.1 (0.1)	4.9 (0.8)
CYS-13	–8.1 (1.3)	13 (2.2)	–0.2 (0.1)	4.6 (1.0)

Table S6b. Residue-wise energy contribution for the 13-mer (GPAGIDGPAGIRC)–OTB (Macrostate 3) complex.

Residue	Non-Bonded (kJ/mol)	Polar (kJ/mol)	Non-Polar (kJ/mol)	Net Energy (kJ/mol)
GLY-1	–0.9 (0.3)	3.6 (1.1)	–0.1 (0.1)	2.7 (0.9)
PRO-2	–1.4 (0.3)	0.5 (0.2)	–0.1 (0.1)	–1.0 (0.3)
ALA-3	–1.4 (0.3)	1.2 (0.3)	–0.1 (0.1)	–0.3 (0.2)
GLY-4	–1.4 (0.4)	1.3 (0.4)	–0.1 (0.1)	–0.2 (0.1)
ILE-5	–2.8 (0.8)	0.8 (0.4)	–0.3 (0.1)	–2.3 (0.6)
ASP-6	–10.6 (2.0)	19.8 (3.5)	–0.4 (0.1)	8.9 (1.6)
GLY-7	–4.0 (0.9)	2.8 (0.6)	–0.2 (0.1)	–1.3 (0.4)
PRO-8	–3.8 (0.7)	1.7 (0.4)	–0.3 (0.1)	–2.4 (0.4)
ALA-9	–3.3 (0.6)	1.6 (0.5)	–0.3 (0.1)	–2.0 (0.4)
GLY-10	–2.3 (0.5)	2.0 (0.5)	–0.2 (0.1)	–0.5 (0.3)
ILE-11	–1.7 (0.5)	1.1 (0.4)	–0.1 (0.1)	–0.7 (0.2)
ARG-12	–3.7 (1.1)	5.5 (1.6)	–0.3 (0.1)	1.5 (0.6)
CYS-13	–1.0 (0.7)	1.4 (1.0)	0 (0.1)	0.4 (0.5)
